# Dynamic Regulation of Supramolecular Chirality in an Amphiphilic Dimeric Macrocycle Through Azobenzene‐Based Photoisomerization

**DOI:** 10.1002/advs.75410

**Published:** 2026-04-22

**Authors:** Minzan Zuo, Yi Shi, Xueqi Tian, Tao Zhang, Jianmin Jiao, Yuhong Shen, Yutong Xie, Jianwen Wei, Xiao‐Yu Hu

**Affiliations:** ^1^ College of Chemistry and Materials Jiangxi Normal University Nanchang China; ^2^ School of Physics Peking University Beijing China; ^3^ College of Materials Science and Technology Nanjing University of Aeronautics and Astronautics Nanjing China; ^4^ Institute of Chinese Medical Sciences University of Macau Taipa China

**Keywords:** chirality regulation, dimeric macrocycle, isomerization, meso‐hybridization, planar chirality

## Abstract

The dynamic control of supramolecular chirality continues to represent a significant challenge in the development of adaptive functional materials. Inspired by dynamic planar chirality modulation and the host‐guest chemistry of pillararenes, we report a stimuli‐responsive supramolecular system capable of reversible chirality switching within an amphiphilic dimeric macrocycle (***m*‐TPE WP5‐PCP**). This hybrid architecture, synthesized via *meso*‐functionalization of pillar[5]arene and [1_5_]paracyclophane, preserves orthogonal host‐guest properties while exhibiting unique amphiphilicity‐directed self‐assembly behavior. Under UV irradiation, *trans*‐to‐*cis* isomerization of an alanine‐substituted azobenzene guest induces stereoselective binding modes, which dynamically modulate the planar chirality and morphologies of ***m*‐TPE WP5‐PCP**. Subsequent removal of the UV stimulus reverses the isomerization process, leading to the reformation of threaded complexes and the restoration of chiral supramolecular assemblies. This process demonstrates hierarchical regulation of supramolecular handedness via orthogonal molecular recognition pathways. The proposed strategy effectively integrates supramolecular chirality switching with stimuli‐responsive energy transduction, offering new avenues for the development of adaptive nanomaterials.

## Graphical abstract

A stimuli‐responsive supramolecular system has been rationally designed enabling hierarchical control over supramolecular handedness. This hybrid architecture retains orthogonal host‐guest properties and displays unique amphiphilicity‐directed chiral self‐assembly.

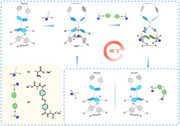

## 1 Introduction

Macrocyclic hosts typically exhibit more symmetric architectures compared to their linear counterparts, primarily due to the absence of terminal functional groups [1, 2, 3, 4, 5]. Among known macrocycles, pillar[n]arenes display particularly high symmetry, resulting from the para‐bridged arrangement of their repeating aromatic units. This structural feature not only confers a well‐defined, rigid pillar‐shaped conformation but also introduces intrinsic planar chirality [6, 7, 8]. In principle, pillar[5]arenes can exist as eight stereoisomeric forms, corresponding to four pairs of enantiomers. However, in practice, steric repulsion or multiple interactions between adjacent subunits usually cause pillar[5]arenes with stable planar chirality to strongly favor a racemate of all‐*p*S and all‐*p*R planar‐chiral configurations. That is, the flipping and swing of pillar[5]arene units are inhibited, and thereby they exhibit stably locked configurations [9, 10, 11, 12]. Moreover, the electron‐rich cavities of pillararenes can undergo inversion, endowing them with dynamic planar chirality. This chirality can be modulated by the noncovalant interactions between the guest molecule and the cavity of pillararenes [13, 14, 15, 16]. This characteristic distinguishes them from other classical macrocycles (e.g., crown ethers, calixarenes, and cyclodextrins) and highlights their promising applicability in the development of chiroptical materials.

Although significant progress has been achieved in regulating the planar chirality of pillararenes, the primary strategies for chirality control in pillararene systems to date have been based on constructing chiral bare macrocycle, pseudo[1]catenane or (pseudo)rotaxanes from either fully hydrophilic or fully hydrophobic derivatives. In contrast, relatively little attention has been paid to regulating the chirality of their further self‐assemblies [17, 18, 19, 20, 21, 22, 23, 24, 25, 26, 27]. Amphiphilic pillararenes play a pivotal role in self‐assembly processes due to their unique capabilities to form responsive and adaptive nanostructures. To date, extensive synthetic efforts have concentrated on modifying the rim regions of the pillararene scaffold to achieve amphiphilicity. On the other hand, although pioneering works on methylene modification of macrocycles have been reported [28, 29, 30, 31, 32, 33, 34, 35], no examples have been reported regarding *meso*‐position modifications using a macrocycle that simultaneously maintain both host–guest binding properties and the structural integrity of the macrocyclic core.

**FIGURE 1:**
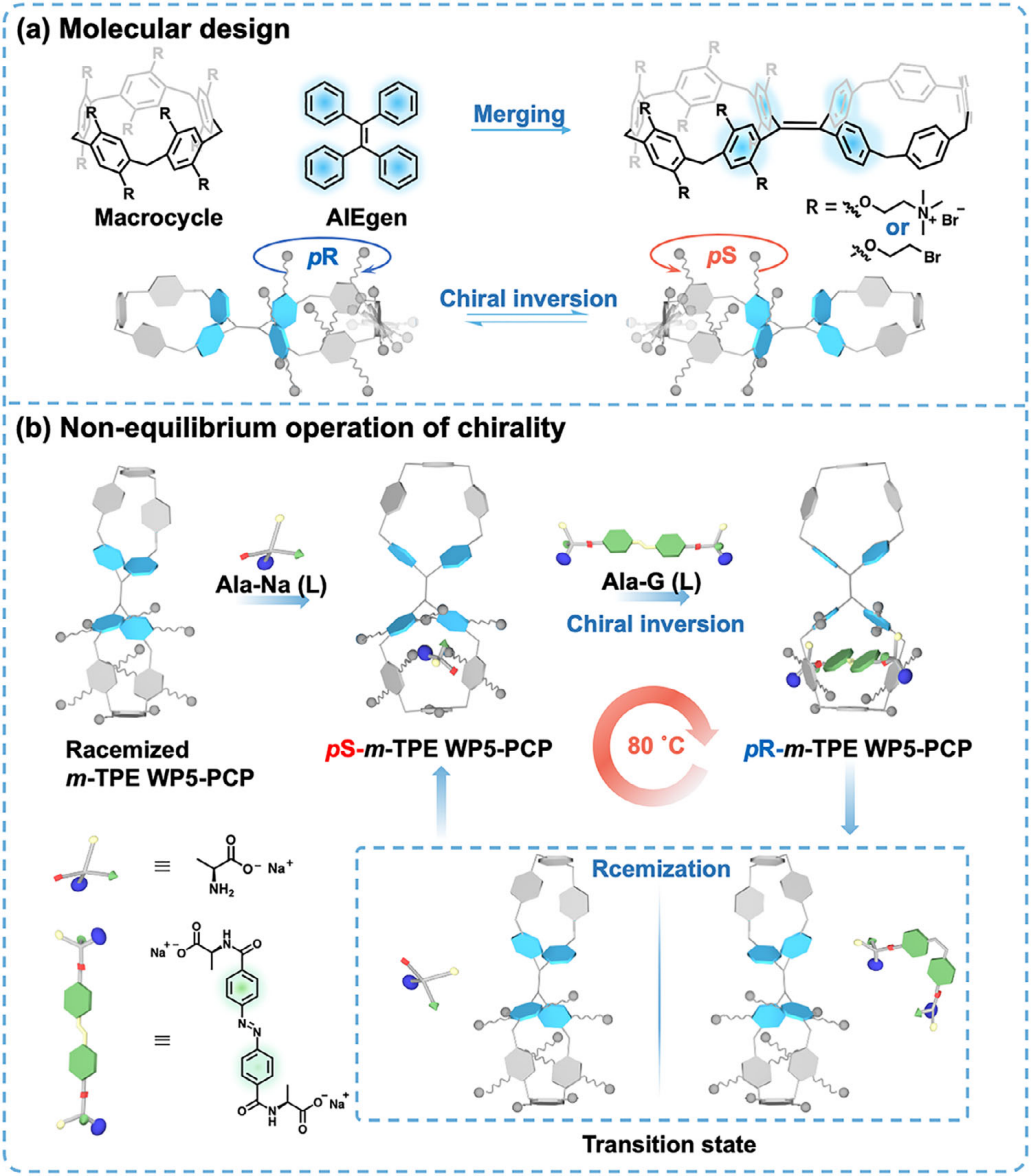
(a) Molecular design of ***m*‐TPE WP5‐PCP**. (b) The photochemical‐fueled process enables sequential programmability of the supramolecular chirality, offering a macrocycle planar chirality switch.

Recently, our group has reported a series of dimeric macrocycles constructed by linking two *meso*‐functionalized host molecules [36, 37]. Considering the complex synthetic procedures involved in the synthesis of such dimeric macrocycles, we envisage that through deliberate and rational molecular design, a hydrophobic unit can be integrated into a hydrophilic pillararene framework. This strategic integration would confer amphiphilic characteristics to the resulting bismacrocycle, thereby enabling control over both the chirality of the pillararene units and the overall supramolecular assembly. In this work, we report the synthesis of an amphiphilic dimeric pillararene, specifically functionalized at the *meso*‐position (Figure 1a). The tetraphenylethylene (TPE)‐embedded water‐soluble pillar[5]arene‐[1_5_]paracyclophane conjugate (***m*‐TPE WP5‐PCP**) exhibits intrinsic amphiphilicity, with the [15]paracyclophane moiety serving as the hydrophobic component and the pillar[5]arene unit functioning as the hydrophilic counterpart. The *meso*‐hybridization of these two distinct macrocyclic compounds preserve their respective structural properties, while giving rise to novel hybrid architectures with unique self‐assembly behavior, thus providing a versatile building block for the construction of diverse hierarchical morphologies.

In addition, leveraging the intrinsic planar chirality of pillararenes, a light‐driven system was developed toward the regulation of ***m*‐TPE WP5‐PCP** chirality, thereby enabling precise control over the chirality of the resulting supramolecular assemblies (Figure 1b). Since [1_5_]paracyclophane and pillar[5]arene demonstrates distinctive host‐guest properties (the [1_5_]paracyclophane lacks host‐guest properties [37], while the pillar[5]arene demonstrates selective recognition toward amino acids [13, 14]), this orthogonal design not only avoids potential competitive binding between the host and guest, but also provides a well‐defined mechanism for subsequent chiral induction of the system. Benefiting from the orthogonal molecular recognition capability of the dimeric macrocycle, the planar chirality of ***m*‐TPE WP5‐PCP** was initially induced through the threading of alanine into the macrocyclic cavity. To achieve the chiral inversion of the system, an alanine‐functionalized azobenzene derivative was introduced, enabling light to serve as an external stimulus to initiate the dynamic process. Upon irradiation with 365 nm UV light, azobenzene underwent isomerization from the *trans* to the *cis* configuration, concurrently inducing racemization of the macrocycle's chiral state. This shift altered the chemical potential of the entire system, driving it away from thermodynamic equilibrium. Upon cessation of UV irradiation, the metastable *cis*‐azobenzene gradually reverted to the more thermodynamically stable *trans* isomer through thermal relaxation, releasing heat to the surroundings and allowing the system to return to equilibrium with restoration of macrocyclic chirality. Notably, the relaxation kinetics of azobenzene are temperature‐dependent; therefore, all experiments were conducted at 80°C to accelerate the dissipation dynamics of the system. This methodology facilitates a sophisticated self‐assembly process featuring transient supramolecular chirality or kinetically controlled self‐assembly with tunable chiroptical properties. The design principle relies on the photo‐responsive modulation of binding affinity between ***m*‐TPE WP5‐PCP** and the chiral guest, reflecting light‐induced conformational transitions.

## 2 Results and Discussion

***m*‐TPE WP5‐PCP** were synthesized via the McMurry coupling reaction between a *meso*‐carbonyl substituted pillar[5]arene and a *para*‐aromatic hydrocarbon (Scheme S1 and Figures S1–S12). Although the precursor compound **11** was treated with excess trimethylamine, steric hindrance around the peripheral reactive sites might prevent the complete quaternization of all side chains. Therefore, the isolated material ***m*‐TPE WP5‐PCP** is more conservatively described as a highly quaternized product containing closely related species with different extents of quaternization, rather than an unambiguously identified single decacationic compound. To explore the dynamic chiral regulation of ***m*‐TPE WP5‐PCP**, α‐amino acid‐functionalized azobenzene derivatives were used as the guest mulecules (Scheme S3 and Figures S13–S21).

**FIGURE 2:**
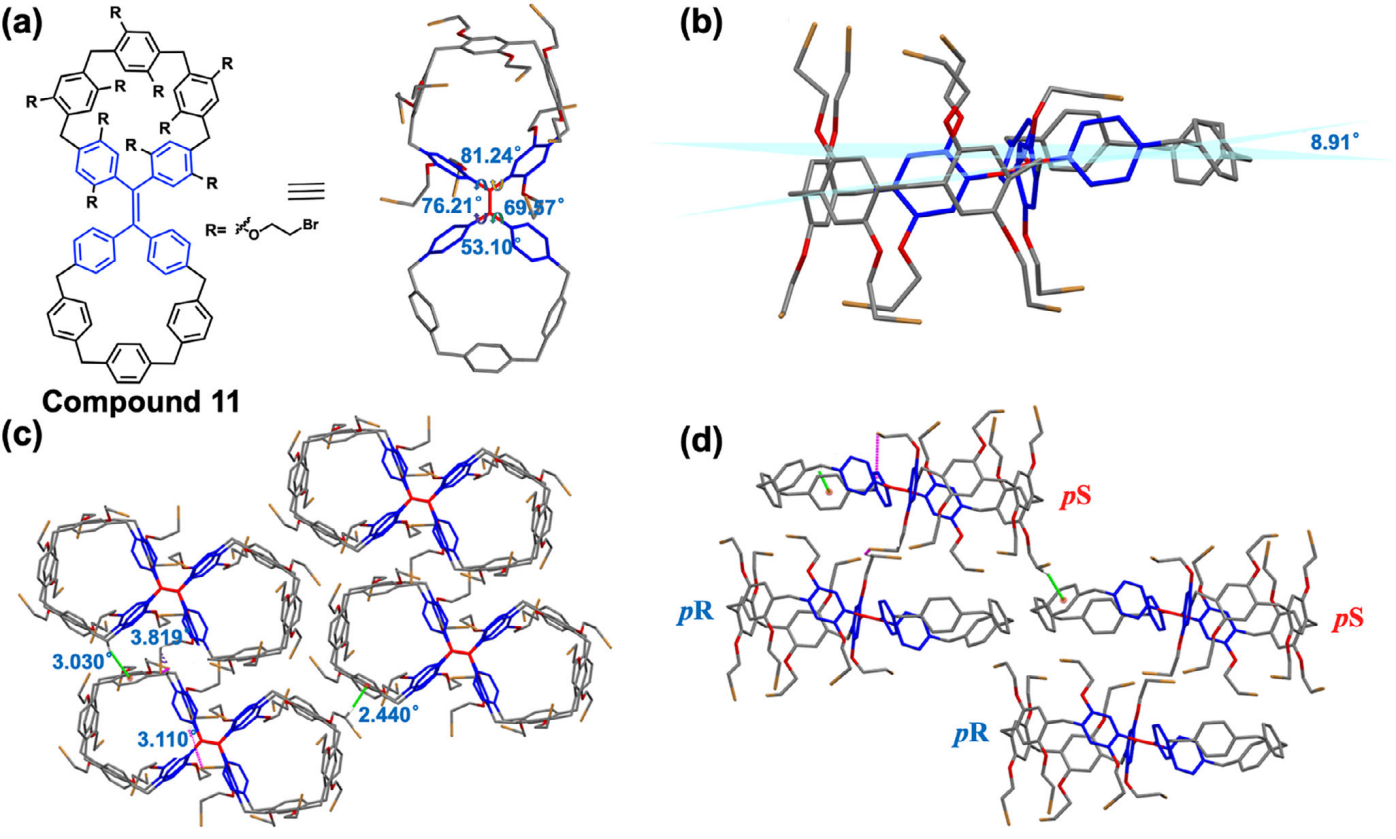
Chemical structure (a), single crystal structure (b), and molecular packing form (c) of compound **11**. Green dashed lines represent C─H···π interactions; Purple dashed lines represent C─H─Br interactions. (d) Two enantiomers (*p*R and *p*S) of compound **11**.

As attempts to obtain a single crystal of ***m*‐TPE WP5‐PCP** were unsuccessful, we shifted our focus to studying the structure of its precursor compound **11**. In this structure, the AIEgen TPE unit is embedded within the dimeric macrocycle. The four dihedral angles between the benzene rings and the C═C bond plane are measured at 69.57°, 81.24°, 53.10°, and 76.21°, respectively. The dihedral angles between the bromoethoxypillararene plane and the [1_5_]PCP plane is 8.91° (Figure 2b), which likely arises from steric hindrance‐induced repulsion between adjacent dimeric macrocycles. The molecular packing of compound **11** reveals that the single crystal structure belongs to the P‐1 space group (Figure 2c), with molecules arranged antiparallelly along the b‐axis. In addition, short C─H···π interactions are observed among three adjacent dimeric macrocycles, with contact distances of 3.030 and 2.440 Å, respectively. C─H─Br contacts are also present, with H─Br distances of 3.110 and 3.189 Å. Notably, crystallographic analysis reveals the coexistence of both *p*R and *p*S macrocyclic conformations within the crystal lattice (Figure 2d; Figure S22b), with the two enantiomers exhibiting mirror‐symmetric characteristics.

Next, the AIE properties of the amphiphilic dimeric macrocycle ***m*‐TPE WP5‐PCP** were investigated. Unlike traditional AIE molecules, ***m*‐TPE WP5‐PCP** showed strong fluorescence in both good solvents, such as water, and poor solvent such as THF, which can be attributed to its self‐aggregation behavior. Moreover, the fluorescence intensity gradually increased with the THF fraction and reached a maximum when the THF content was 90% (Figure S23). The critical aggregation concentration for constructing supramolecular nanoaggregates was further determined to be 38.4 µM through fluorescent titration experiments (Figure S24). Transmission electron microscopy (TEM) analysis revealed that ***m*‐TPE WP5‐PCP** self‐assemblies formed spherical particles with an average diameter of approximately 78 nm (Figure S25).

**FIGURE 3:**
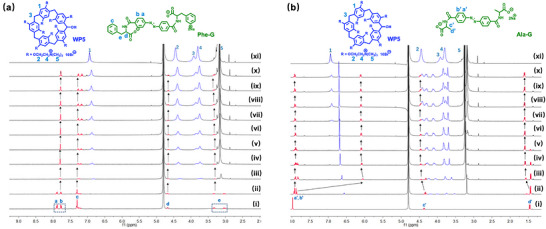
(a) ^1^H NMR spectra of **Phe‐G** (D_2_O, 400 MHz, and 298 K) in the presence of increasing equivalents of **WP5**; from (i) to (x): 0, 0.1, 0.3, 0.6, 0.8, 1, 1.3, 1.6, 1.8, and 2.0 equivalents. xi) free **WP5**. (b) ^1^H NMR spectra of **Ala‐G** (D_2_O, 400 MHz, and 298 K) in the presence of increasing equivalents of **WP5**; from (i) to (x): 0, 0.1, 0.3, 0.6, 0.8, 1, 1.3, 1.6, 1.8, and 2.0 equivalents. xi) free **WP5**.

Subsequently, alanine‐ and phenylalanine‐functionalized azobenzene derivatives were used as the guests to develop supramolecular nanoaggregates with tunable chirality transformation properties. As indicated in our previous reports [37], [1_5_]paracyclophane lacks distinctive host‐guest properties, whereas pillar[5]arene demonstrates selective recognition toward amino acids. Therefore, only the interactions between the guest molecules and the pillar[5]arene moiety were considered in this study. To facilitate a more straightforward analysis, the hydrophilic host **WP5** was selected as a model system. Initially, the interactions between **WP5** and phenylalanine‐derived azobenzene (**Phe‐G**) (1:1) were examined by ^1^H NMR titrations. A fast exchange regime between **Phe‐G** and **WP5** was observed on the NMR timescale (Figure 3a). Upon addition of **WP5**, the H_a_ and H_b_ signals of the free **Phe‐G** gradually merged into a single peak, without a significant shielding effect. Meanwhile, the H_c_ signals split into two peaks, indicating that its carboxylic salt group enters the cavity of **WP5**. The 1:1 binding stoichiometry was further confirmed by the Job's plot method (Figure S28). In contrast, the interaction between **WP5** and **Ala‐G** exhibited a slow exchange regime (Figure 3b). Upon addition of **WP5**, the H_a’_ and H_b’_ peaks of free **Ala‐G** split into two peaks and shifted to the upfield region, suggesting that the azobenzene moiety may thread into the electron‐rich cavity of **WP5**. In addition, the H_d’_ signal of the free guest completely disappeared after the addition of 1.0 equivalents of **WP5**, further supporting a 1:1 binding ratio between **Ala‐G** and **WP5**.

To validate these observations, 2D NOESY NMR experiments were performed by using **Phe‐G**/**Ala‐G** and ***m*‐TPE WP5‐PCP** in an equimolar solution in D_2_O. For **Phe‐G**, NOE correlations were observed between H_1_ of **WP5** and H_b_ of **Phe‐G**, indicating that H_b_ is spatially close to the cavity of **WP5** (Figure S26). Moreover, due to the size limitation of the **WP5** cavity, only the carboxylic salt group can enter the cavity of **WP5**, while the benzene ring remains outside. For **Ala‐G**, NOE correlations were observed between H_1_ of **WP5** and H_a’_ of **Ala‐G**, as well as between H_3_ of **WP5** and H_c’_ of **Ala‐G**, confirming that the azobenzenes moiety of **Ala‐G** is capable of threading into the cavity of **WP5** to form a (pseudo)rotaxane structure, as shown in Figure S27.

**FIGURE 4:**
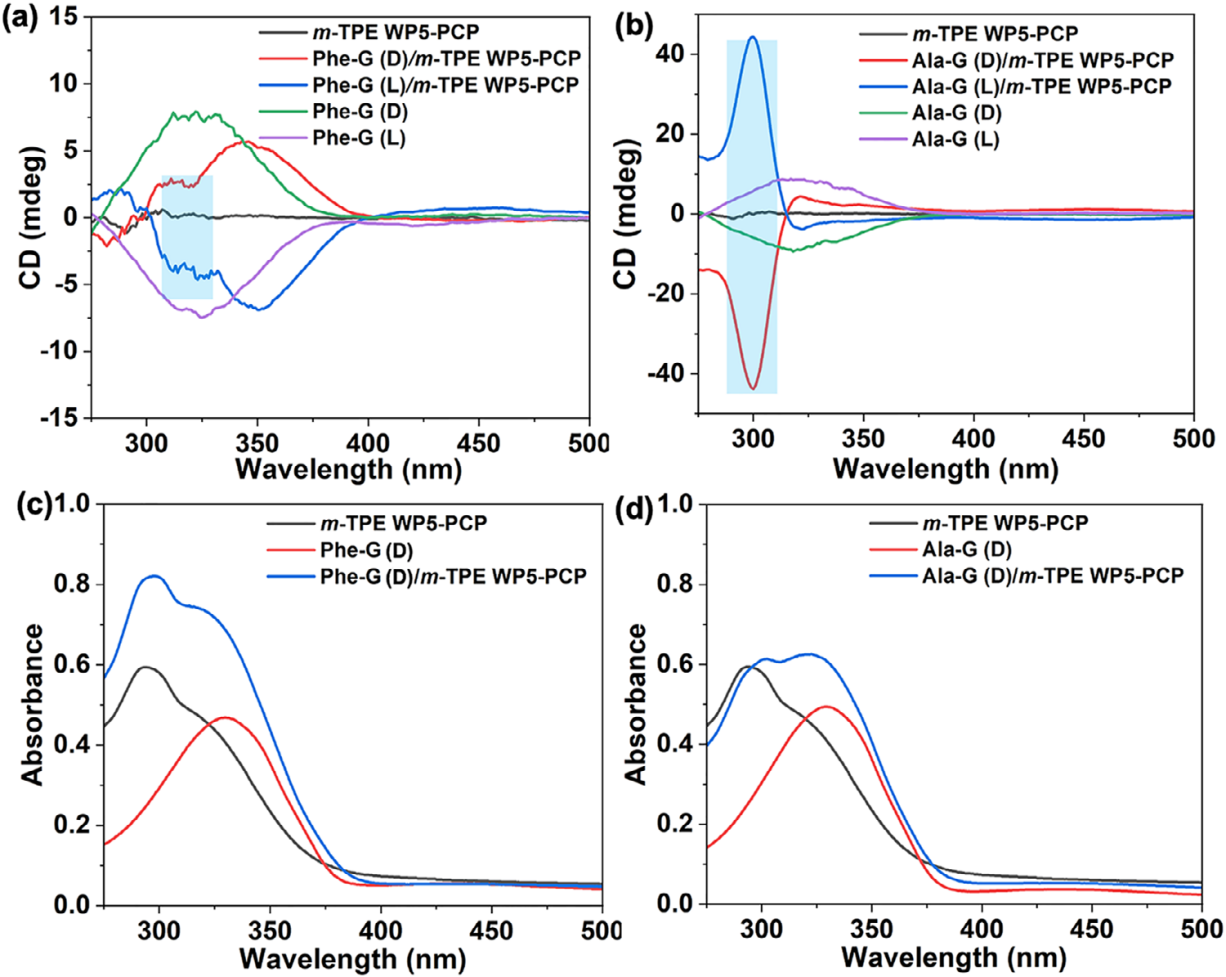
(a) CD spectra of ***m*‐TPE WP5‐PCP**, **Phe‐G** (D/L), and **Phe‐G** (D/L)/***m*‐TPE WP5‐PCP** in water. (b) CD spectra of ***m*‐TPE WP5‐PCP**, **Ala‐G** (D/L), and **Ala‐G** (D/L)/***m*‐TPE WP5‐PCP** in water. (c) CD spectra of ***m*‐TPE WP5‐PCP**, **Phe‐G**, and **Phe‐G**/***m*‐TPE WP5‐PCP** in water. (d) CD spectra of ***m*‐TPE WP5‐PCP**, **Ala‐G**, and **Ala‐G**/***m*‐TPE WP5‐PCP** in water.

Considering the intrinsic chirality of **Ala‐G** and **Phe‐G**, the chiral conformations of ***m*‐TPE WP5‐PCP** induced by these guests were studied using CD spectroscopy. ***m*‐TPE WP5‐PCP** showed a negligible CD signal due to its dynamically racemic planar chirality in solution, whereas **Phe‐G** (D/L) displayed strong positive/negative cotton effects at 325 nm (Figure 4a). Upon the addition of 1.0 equiv. of **Phe‐G** (D/L) to the ***m*‐TPE WP5‐PCP** solution, bisignate CD bands, positive signal at 300 nm, and the negative signal at 290 nm were observed, corresponding to the absorption signal of ***m*‐TPE WP5‐PCP**. In contrast, **Ala‐G** (D/L) displayed strong negative/positive Cotton effects at 325 nm (Figure 4b). Moreover, mirrored Cotton effects at 300 nm, corresponding to the absorption at 299 nm, were observed for **Ala‐G** (D)/***m*‐TPE WP5‐PCP** (negative) and **Ala‐G** (L)/***m*‐TPE WP5‐PCP** (positive). These results reveal the formation of opposite planar chiral configurations within the **WP5** moiety, confirming that the different side chains of the substituted amino acids enter the cavity and modulate the host's conformational arrangement.

**FIGURE 5:**
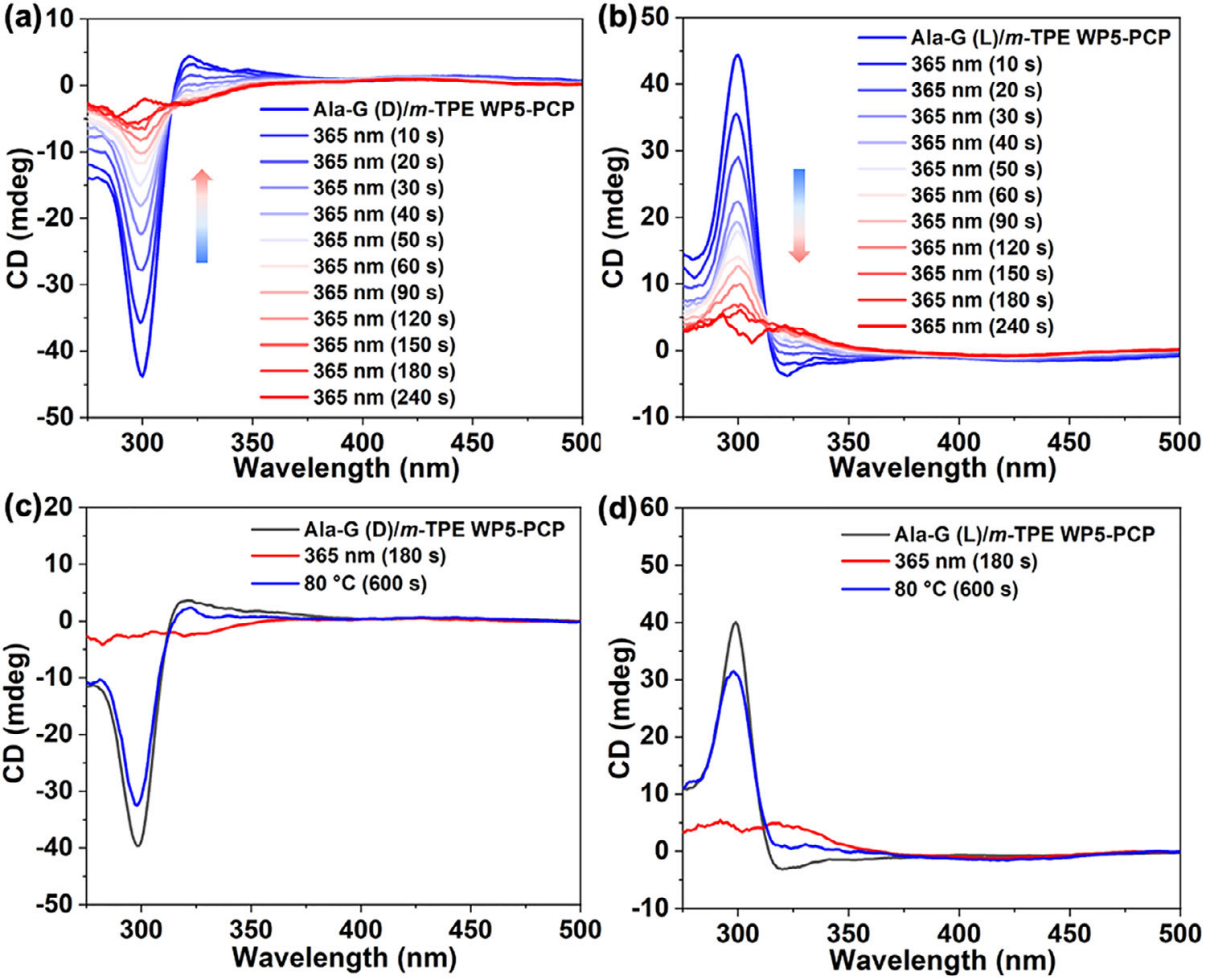
(a) Time‐dependent CD spectra of **Ala‐G** (D)/***m*‐TPE WP5‐PCP** in water upon UV light irradiation. (b) Time‐dependent CD spectra of **Ala‐G** (L)/***m*‐TPE WP5‐PCP** in water upon UV light irradiation. (c) CD spectral changes of **Ala‐G** (D)/***m*‐TPE WP5‐PCP** in water under irradiation at 365 nm and heating at 80°C. (d) CD spectral changes of **Ala‐G** (L)/***m*‐TPE WP5‐PCP** in water under irradiation at 365 nm and heating at 80°C.

Since the binding ability would affects the strength of the induced Cotton effect, the slow‐changing guest **Ala‐G**, which exhibits stronger noncovalent interaction, was selected for further investigation. To avoid any potential influence of **Ala‐G** guest on the host‐guest complex, the chiral changes of the individual **Ala‐G** molecule were initially examined. UV irradiation did not result in significant changes in the Cotton effects corresponding to the UV absorbance of the azobenzene group (320 nm), likely because UV light induces only the *cis/trans* configurational transition of the azobenzene group, rather than altering its chirality (Figure S29a,b). Furthermore, the CD signals were observed to recover upon heating the system, suggesting the reversible *cis*‐to‐*trans* isomerization of the azobenzene group (Figure S29c,d). Time‐dependent UV spectra of **Ala‐G** alone showed that, upon irradiation at 365 nm, the characteristic absorption peak of azobenzene at 330 nm rapidly decreased over 300 s and fully recovered after heating at 80°C for 25 min, further reflecting its *cis*‐to‐*trans* isomerization (Figure S30). Subsequently, the reversible chiral changes of **Ala‐G**/***m*‐TPE WP5‐PCP** (pseudo)rotaxane were investigated. Upon UV light irradiation, the CD signal corresponding to the UV absorbance of the ***m*‐TPE WP5‐PCP** group (300 nm) gradually decreased over a period of 180 s. Notably, a signal inversion was observed upon prolonged irradiation, manifesting as mirrored Cotton effects at 300 nm (Figure 5a,b). These observations suggest that UV irradiation induces photoisomerization of the azobenzene unit from the *trans* to the *cis* configuration, thereby promoting dethreading of the (pseudo)rotaxane structure and resulting in the ***m*‐TPE WP5‐PCP** adopting a racemic conformation. Furthermore, the planar chirality of ***m*‐TPE WP5‐PCP** can be reversed upon thermal treatment, which is attributed to the reformation of the threaded complex between *trans*‐**Ala‐G** and ***m*‐TPE WP5‐PCP** (Figure 5c,d).

**FIGURE 6:**
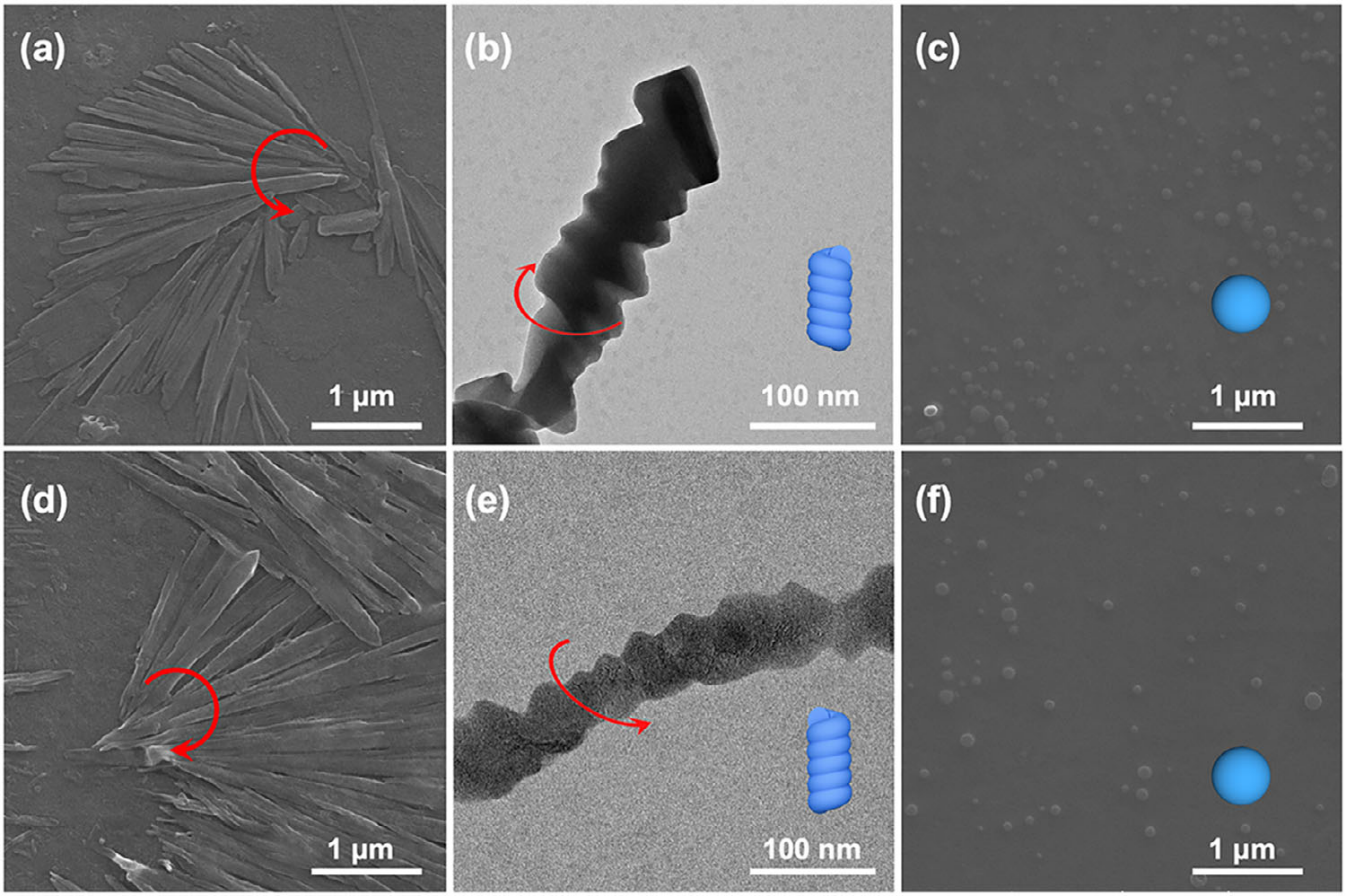
(a) SEM image of **Ala‐G** (D) self‐assemblies. (b) TEM image of **Ala‐G** (D)/***m*‐TPE WP5‐PCP** self‐assemblies. (c) SEM image of **Ala‐G** (D)/***m*‐TPE WP5‐PCP** self‐assemblies upon irradiation at 365 nm. (d) SEM image of **Ala‐G** (L) self‐assemblies. (e) TEM image of **Ala‐G** (L)/***m*‐TPE WP5‐PCP** self‐assemblies. (f) SEM image of **Ala‐G** (L)/***m*‐TPE WP5‐PCP** self‐assemblies upon irradiation at 365 nm.

Meanwhile, the morphologies of **Ala‐G** and **Ala‐G**/***m*‐TPE WP5‐PCP** were investigated using scanning electron microscopy (SEM) and transmission electron microscope (TEM). The results demonstrate that **Ala‐G** (D) and **Ala‐G** (L) self‐assemble into anti‐clockwise and clockwise twisted nanoflakes in aqueous solution, respectively, with lengths extending to several micrometers and widths of approximately 200 nm (Figure 6a,c). In the case of the **Ala‐G**/***m*‐TPE WP5‐PCP** (pseudo)rotaxane systems, they preferentially assemble into uniform helical nanostructures, wherein the handedness of the nanotwist is dictated by the chirality of the (pseudo)rotaxane. Specifically, the D‐configured guest induces a defined planar chirality in ***m*‐TPE WP5‐PCP**, resulting in left‐handed helical nanotubes. In contrast, the L‐configured guest induces an opposite planar chirality, leading to right‐handed helical nanotubes (Figure 6b,e). Upon irradiation with 365 nm light, the **Ala‐G**/***m*‐TPE WP5‐PCP** assemblies undergo a structural transformation into spherical nanostructures with an average particle size of approximately 70 nm (Figure 6c,f).

**FIGURE 7:**
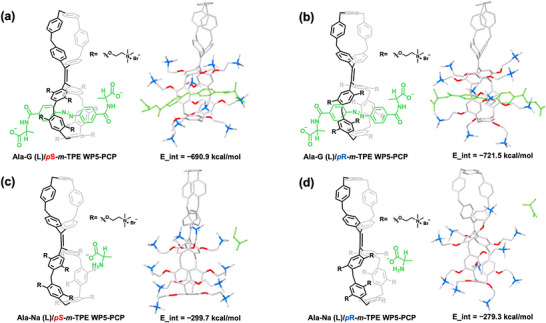
Free energies and optimized structures of (a) **Ala‐G** (L)/*p*S‐***m*‐TPE WP5‐PCP**, (b) **Ala‐G** (L)/*p*R‐***m*‐TPE WP5‐PCP**, (c) **Ala‐Na** (L)/*p*S‐***m*‐TPE WP5‐PCP**, and (d) **Ala‐Na** (L)/*p*R‐***m*‐TPE WP5‐PCP**. Hydrogens of the complexes are omitted for clarity.

To further elucidate the chirality induction effects exerted by guest molecules on the dimeric macrocycle, molecular dynamics (MD) simulations and density functional theory (DFT) calculations were conducted. Here, for the purpose of facilitating calculation and comparison, we selected the idealized ***m*‐TPE WP5‐PCP** bearing ten ammonium groups for subsequent theoretical calculation experiments. The computational analysis quantified the thermodynamic preference between diastereomeric host‐guest complexes. The results reveal a negligible variance (G = 0.0004 kcal/mol) in Gibbs free energy between the *p*R‐ and the *p*S‐***m*‐TPE WP5‐PCP** enantiomers, with respective values of −5974.4058 and −5974.4062 kcal/mol (Figure S31 and Table S2), consistent with the thermodynamic equivalence characteristic of enantiomeric pairs. For the (pseudo)rotaxane system, a significant energetic stabilization of 30.6 kcal/mol was observed for the **Ala‐G** (L)/*p*R‐***m*‐TPE WP5‐PCP** complex compared to its **Ala‐G** (L)/*p*S‐***m*‐TPE WP5‐PCP** counterpart (Figure 7a,b; Figure S32 and Table S3) [38], which correlates with the experimentally observed positive Cotton effect in the CD spectra—a characteristic signature of the *p*R‐configuration in pillar[5]arene derivatives, as previously reported [39, 40]. Furthermore, to validate the influence of different amino terminals on the chirality of pillararenes, we independently investigated the binding modes and binding energies between sodium alanine (**Ala‐Na**) and the pillar[5]arene enantiomers. The **Ala‐Na** (L)/*p*S‐***m*‐TPE WP5‐PCP** complex is 20.4 kcal/mol more stable than the **Ala‐Na** (L)/*p*R‐***m*‐TPE WP5‐PCP** complex (Figure 7c,d; Table S3), which aligns well with the observation that *p*S‐pillar[5]arene tends to exhibit a negative CD signal. This stereoselective complexation originates from the differing chiral complementarity between the guest molecules and the macrocyclic hosts [41]. The energetically favored **Ala‐G** (L)/*p*R‐***m*‐TPE WP5‐PCP** complex demonstrates optimal spatial congruence, requiring minimal geometric distortion of the macrocycle upon encapsulation. Conversely, the disfavored **Ala‐G** (L)/*p*S‐***m*‐TPE WP5‐PCP** complex manifests geometric strain, necessitating additional energy input to overcome the rotational barriers in the ***m*‐TPE WP5‐PCP** host to enable accommodation.

To further invstigate the helical handedness bias of the host/guest complex, we extended the theoretical calculations to include a tetramer system. Adjacent *p*S‐***m*‐TPE WP5‐PCP** molecules engage in multiple π‐π stacking and C─H···π interactions, with interatomic distance ranging from 2.560–3.499 Å (Figure S33a). To minimize steric hindrance, neighboring molecules adopt a staggered arrangement within the supramolecular layered structure stacked along the axis, resulting in an interleaved helical structure (Figure S33b). The mechanism for helical handedness can be explained as follows: In an aqueous environment, the hydrophobic segments tend to “escape” from the water phase and aggregate with one another. This hydrophobic effect forces the entire molecular chain to fold or twist, providing the initial driving force for the formation of helix conformations. Concurrently, non‐covalent interactions between molecules (hydrogen bonding, hydrophobic forces, and electrostatic interactions) guide and stabilize the orientation of complexes, leading to the lowest‐energy and most stable conformation. Furthermore, since each molecular unit is pre‐organized into a right‐handed helix, when they approach one another, they arrange according to the homochiral matching principle to maximize intermolecular interactions and minimize steric hindrance. This alignment directly transfers and amplifies the chirality of the individual units, resulting in a macroscopic right‐handed helical morphology of the entire assembled structure.

**FIGURE 8:**
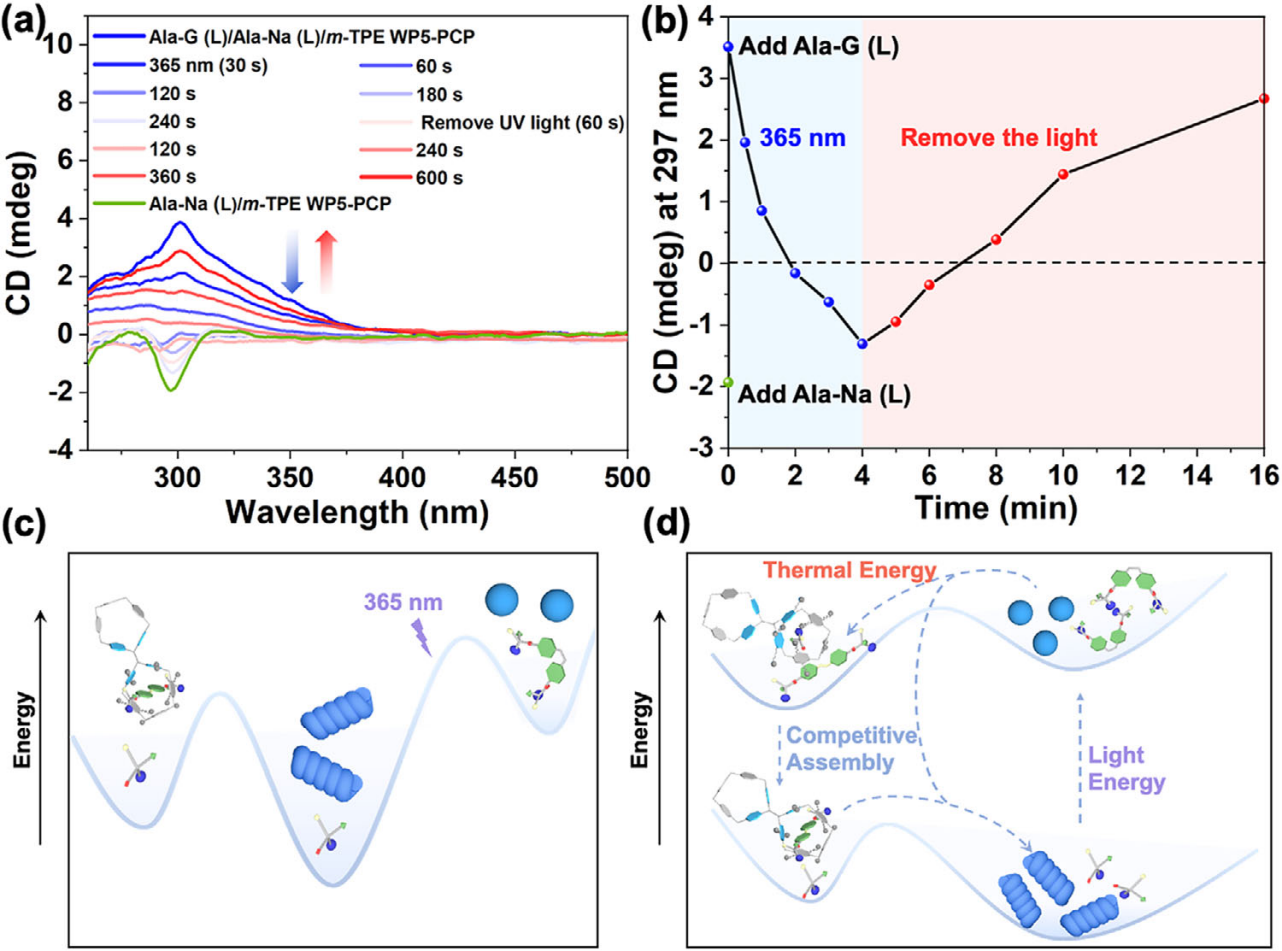
(a) Time‐dependent CD spectra of **Ala‐G** (L)/**Ala‐Na** (L)/***m*‐TPE WP5‐PCP** system upon UV light irradiation and removing the light. (b) Changes of time‐dependent CD spectra of **Ala‐G** (L)/**Ala‐Na** (L)/***m*‐TPE WP5‐PCP** system at 297 nm. (c) Schematic representation of the energy variation of the light induced chiral regulation process. (d) Schematic representation of the energy variation in the chiral regulation of **Ala‐G** (L)/**Ala‐Na** (L)/***m*‐TPE WP5‐PCP** system.

Building upon the aforementioned findings that distinct binding modes (toward/outward) of amino acids with macrocycles can induce varied macrocyclic chirality, we introduced an additional guest molecule, **Ala‐Na**, to construct a disequilibrium supramolecular system based on the ***m‐*TPE WP5‐PCP** macrocycle. The chirality of this disequilibrium system was monitored using circular dichroism spectroscopy (Figure 8a,b). Given that the relaxation time of azobenzene is temperature‐dependent and equilibration may take several days under ambient conditions, the entire process was conducted in an 80°C environment to accelerate the dissipation kinetics. Isothermal titration calorimetry (ITC) data showed that the association constant for **Ala‐G** (L)/***m*‐TPE WP5‐PCP** is 9.2 × 10^4^ M^−1^, while the association constant for **Ala‐Na** (L)/***m*‐TPE WP5‐PCP** is 1.6 × 10^4^ M^−1^ (Figure S34). To excludes the possible interference of the CD signal of the guest *cis* **‐Ala‐G**(L), we adjusted the molar ratio of **Ala‐Na** to be in large excess (molar ratio of **Ala‐Na**/**Ala‐G(**L) = 10/1, a condition that does not significantly affect the competitive binding of the *trans* **Ala‐G(**L) to the host ***m*‐TPE WP5‐PCP**). Initial complexation of **Ala‐Na** (L) with ***m*‐TPE WP5‐PCP** resulted in a negative Cotton effect at 297 nm, consistent with a configuration in which the carboxyl terminus of the guest **Ala‐Na** binds with the macrocyclic cavity. Upon subsequent addition of the competitive guest **Ala‐G** (L), its azobenzene moiety competitively threaded into the ***m*‐TPE WP5‐PCP** cavity, displacing the carboxyl terminus of **Ala‐Na** (L) and orienting the alanine moiety of **Ala‐G** (L) outward from the cavity. This structural reorganization induced a chirality inversion in the ***m*‐TPE WP5‐PCP** macrocycle. Upon irradiation at 365 nm, the CD signal at 297 nm underwent a rapid inversion, transitioning from positive to negative within approximately 240 s. This transition corresponds to *cis*‐isomerization of the azobenzene unit in **Ala‐G** (L), which facilitates the dethreading of the supramolecular (pseudo)rotaxane structure and subsequent rebinding of **Ala‐Na** and free ***m*‐TPE WP5‐PCP**. Notably, removing the UV light for more than 600 s induced a gradual reformation of the (pseudo)rotaxane structure through *trans‐*azobenzene isomerization, accompanied by a corresponding reversal of the CD signal. In comparison to the reversible isomerization dynamic processes, the binding affinity between **Ala‐G** (L) and ***m*‐TPE WP5‐PCP** was stronger than that between **Ala‐Na** and ***m*‐TPE WP5‐PCP**, indicating that the **Ala‐G** (L)/***m*‐TPE WP5‐PCP** system within the supramolecular (pseudo)rotaxane possesses a lower energy state (Figure 8c,d). Therefore, we successfully established chirality switching within the ***m‐*TPE WP5‐PCP** supramolecular system, sustained by a controlled energy cycle involving both photochemical and thermal stimuli. In addition, since both *cis* and *trans* isomers of azobenzene are photoreactive and exhibit significantly overlapping absorption spectra, irradiation at 365 nm can induce both *cis‐*to‐*trans* and *trans‐*to‐*cis* isomerization. As a result, the system can autonomously cycle under continuous illumination [42].

## 3 Conclusion

In summary, we have successfully developed a stimuli‐responsive system that enables dynamic control over supramolecular chirality through tunable planar chirality of pillararenes. The amphiphilic dimeric macrocycle ***m*‐TPE WP5‐PCP** preserves its intrinsic host‐guest properties while exhibiting unique self‐assembly behavior. Driven by stereoselective binding with the alanine‐substituted azobenzene guest **Ala‐G** and the complementary guest **Ala‐Na**, *trans*‐to‐*cis* isomerization of **Ala‐G** can be induced through UV irradiation, leading to its dethreading from the macrocycle cavity and the programmable chirality inversion of ***m*‐TPE WP5‐PCP**. Meanwhile, removing the UV light reforms the threaded complexes and regenerates the chiral supramolecular assemblies. This process concurrently modulates hierarchical morphologies, enabling adaptive transformation between helical nanotubes and spherical nanoparticles. This strategy not only advances the dynamic control of supramolecular handedness but also offers versatile pathways for developing adaptive nanomaterials, such as transient chiral sensors or reprogrammable nanocarriers.

## 4 Experimental Section

### 4.1 Procedures for Dynamic Regulation of Supramolecular Chirality

In an 80°C environment, **Ala‐Na** (L) was added to a solution of ***m*‐TPE WP5‐PCP**. Subsequently, another portion of Ala‐G (L) was added, and the mixture was irradiated under 6 W (365 nm) light.

Synthesis and relevant characterization details are provided in the Supporting Information.

## Conflicts of Interest

The authors declare no conflict of interest.

## Supporting information




**Supporting File**: advs75410‐sup‐0001‐SuppMat.docx.

## Data Availability

The data that support the findings of this study are available in the supplementary material of this article.
