# Supplementary material for: Dynamic Regulation of Supramolecular Chirality in an Amphiphilic Dimeric Macrocycle Through Azobenzene‐Based Photoisomerization
Source: Adv Sci (Weinh). 2026 Apr 22;13(40):e75410. doi: 10.1002/advs.75410 (PMC13335635; doi:10.1002/advs.75410)
Supplement: Supplementary file 1 — Supporting File: advs75410‐sup‐0001‐SuppMat.docx. [file ADVS-13-e75410-s001.docx]

**Supporting Information**

**Dynamic Regulation of Supramolecular Chirality in an Amphiphilic Dimeric Macrocycle through Azobenzene-Based Photoisomerization**

Minzan Zuo,^†,#^ Yi Shi,^‡,#^ Xueqi Tian,^†,#^ Tao Zhang,^‖,^ Jianmin Jiao,^†^ Yuhong Shen,^‖^ Yutong Xie,^‖^ Jianwen Wei,^¶^ Xiao-Yu Hu^†,‖,*^

^†^College of Chemistry and Materials, Jiangxi Normal University, Nanchang 330022, China.

^‡^School of Physics, Peking University, Beijing, 100871, China.

^‖^College of Materials Science and Technology, Nanjing University of Aeronautics and Astronautics, Nanjing 211106, China.

*^¶^*Institute of Chinese Medical Sciences, University of Macau, Taipa, 999078, China.

**Table of Contents**

1. **Supplementary Methods** 3

General information 3

Synthesis of host molecule ***m-*TPE WP5-PCP** 4

Synthesis of guest molecule 16

2. **Supplementary Discussion** 22

Single crystal structures of ***m-*TPE WP5-PCP** 22

Host/guest interaction of ***m*-TPE WP5-PCP** 24

Dynamic chiral regulation 27

3. **Supplementary Reference** 32

1. **Supplementary Methods**

**General information**

All commonly available reagents and solvents were purchased from Energy Chemical Reagent Co., Ltd. without further purification. Column chromatography was performed with silica gel (200‒300 mesh) produced by Shanghai Titan Scientific Co., Ltd. All yields were given as isolated yields. NMR spectra were recorded on a Bruker AV400 instrument. High-resolution electrospray ionization mass spectra (HR-ESI-MS) were recorded on an Agilent 6540Q-TOF LCMS equipped with an electrospray ionization (ESI) probe operating in the positive-ion mode with direct infusion. UV/vis spectra were recorded on a Shimadzu UV-1700 spectrophotometer. Transmission electron microscope (TEM) investigations were carried out on a FEI Talos F200X instrument. Scanning electron microscope (SEM) investigations were carried out using a FEI Quanta FEG 250 instrument. The crystal structures were determined by single-crystal X-ray analysis. Data collections were performed using a Bruker Apex Smart CCD diffractometer. Circular dichroism spectra were measured on a JASCO J-810 spectrometers using a quartz cuvette of 1 cm path length.

**Synthesis of host molecule *m-*TPE WP5-PCP**


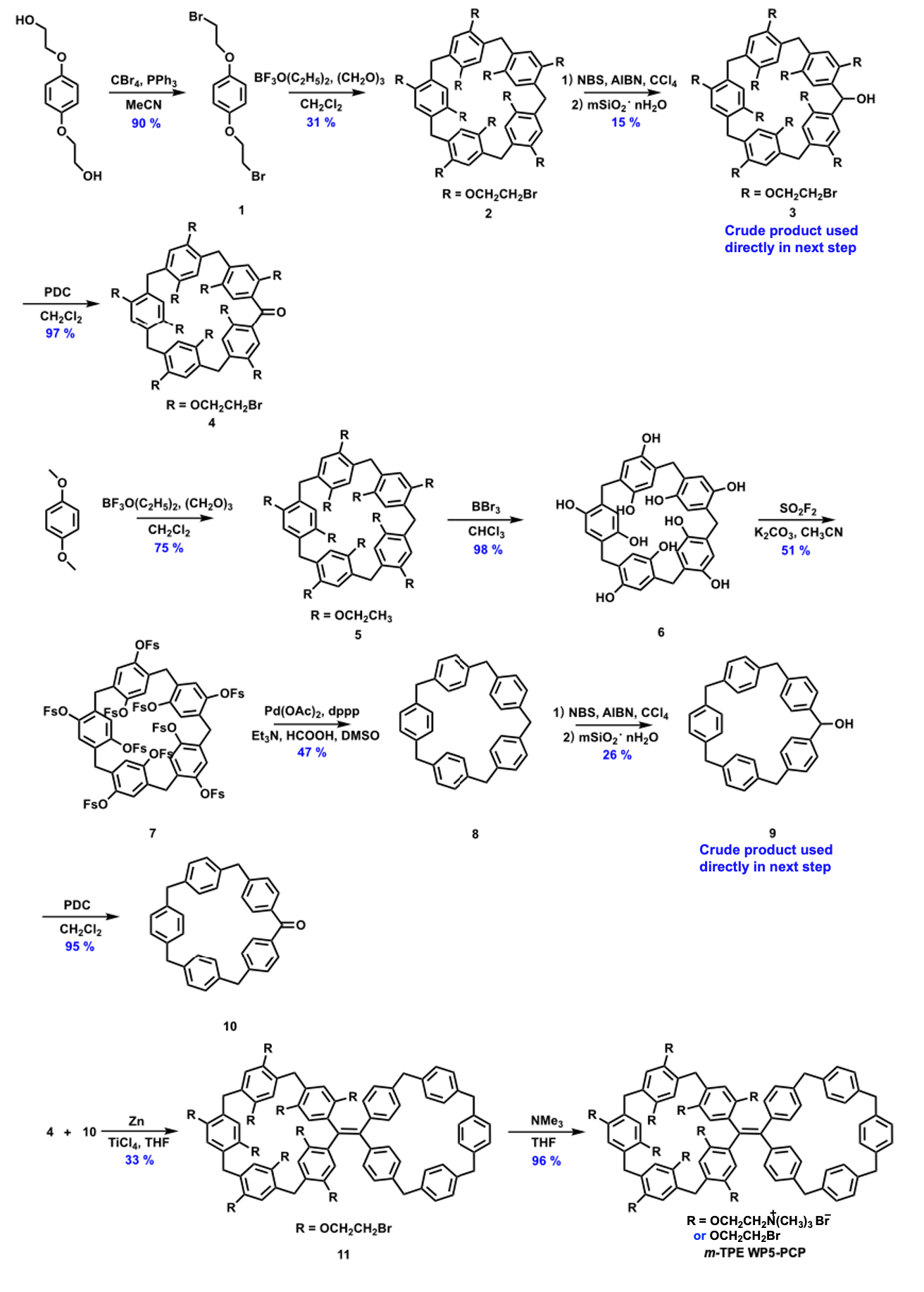


**Scheme S1.** Synthesis route of compound ***m*-TPE WP5-PCP**. ^[S1-S7]^

**Synthesis of compound** **1**^[S1]^

1,4-bis (2-hydroxyethoxy) benzene (3.97 g, 20.0 mmol), triphenylphosphine (15.7 g, 60.0 mmol) and dry acetonitrile (100 mL) were added to a 250 mL three-necked flask under a nitrogen atmosphere. Under vigorous stirring, carbon tetrabromide (19.9 g, 60.0 mmol) was added slowly at 0 °C and the mixture was stirred at room temperature overnight. 50 mL water was added to quench the reaction. Then white precipitation was collected by vacuum filtration, washed with methanol/water (*v/v* = 3/2, 3 × 100 mL), recrystallized from methanol, and dried under vacuum to afford compound **1** as a white crystal (6.10 g, 18.0 mmol, 90%).^1^H NMR (400 MHz, CDCl_3_, 298 K) δ 6.86 (s, 4H), 4.25 (t, *J* = 6.3 Hz, 4H), 3.62 (t, *J* = 6.3 Hz, 4H).


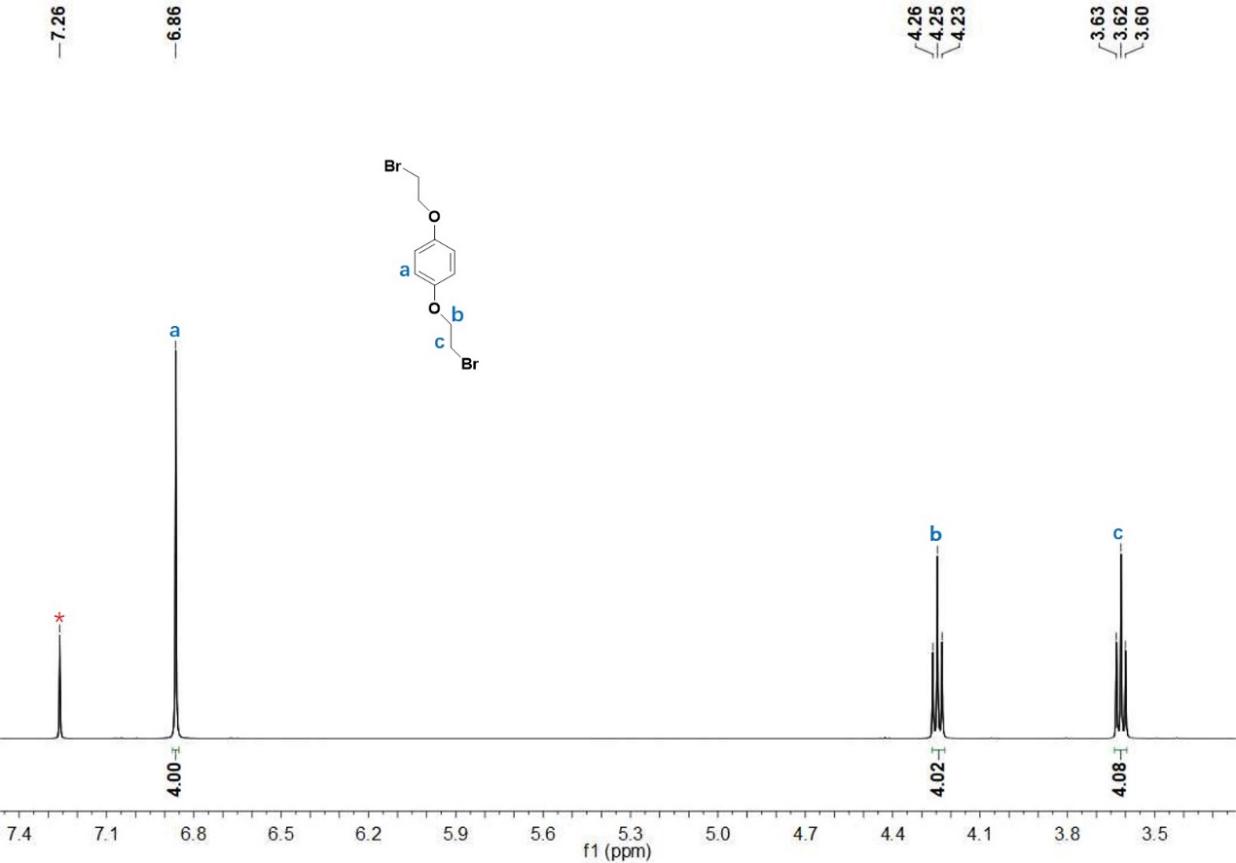


**Figure S1.** ^1^H NMR spectrum (400 MHz, CDCl_3_, 298 K) of compound **1** (* refers to solvent peak).

**Synthesis of compound 2**^[S2]^

Compound **1** (3.89 g, 12.0 mmol), trioxymethylene (1.30 g, 12.0 mmol) and dichloromethane (300 mL) were added to a 1000 mL three-necked flask under nitrogen atmosphere, followed by stirring. Boron trifluoride diethyl etherate (1.5 mL ,12.0 mmol) was added to the solution at 0 °C and the mixture was stirred at room temperature overnight. 100 mL water was added to quench the reaction. Then the aqueous phase was extracted with dichloromethane, the organic phase was dried over anhydrous sodium sulfate, and purified by column chromatography (silica gel, petroleum ether/ dichloromethane = 2:1, *v*/*v*) to obtain compound **2** as a white solid (1.26 g, 0.75 mmol, 31%). ^1^H NMR (400 MHz, CDCl_3_, 298 K) δ 6.91 (s, 10H), 4.23 (t, *J* = 5.6 Hz, 20H), 3.84 (s, 10H), 3.63 (t, *J* = 5.6 Hz, 20H).


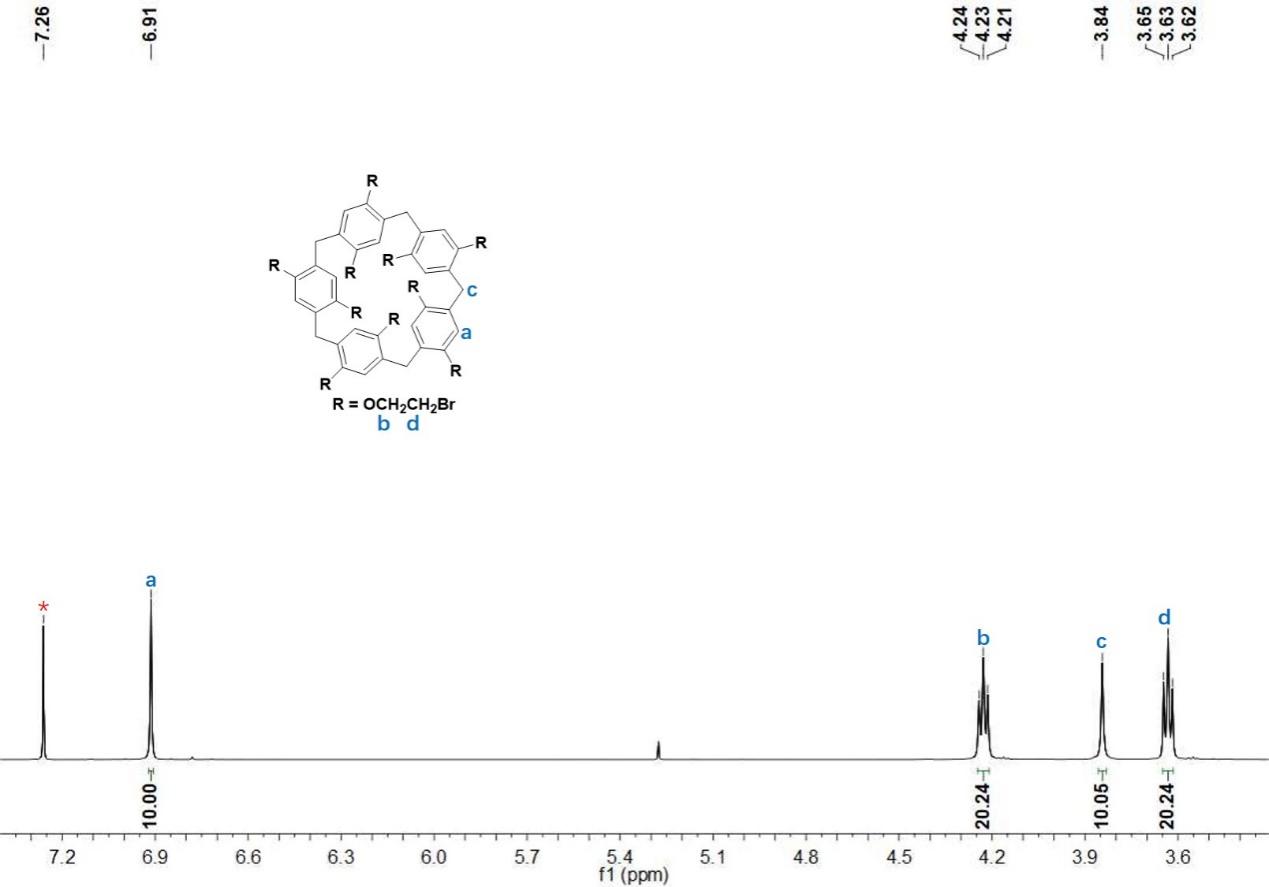


**Figure S2.** ^1^H NMR spectrum (400 MHz, CDCl_3_, 298 K) of compound **2** (* refers to solvent peak).

**Synthesis of compound** **3** **and 4**^[S3,S4]^

Compound **2** (3.00 g, 1.79 mmol) and azodiisobutyronitrile (AIBN, 0.01 g, 0.05 mmol) were dissolved in CCl_4_ (45 mL) and then transferred to a 100 mL three-necked flask. The mixture was stirred and heated to reflux under a nitrogen atmosphere. And *N*-bromosuccinimide (NBS, 0.38 g, 2.14 mmol) dissolved in CCl_4_ (5 mL) was slowly added into the reaction mixture within 6 h. The mixture was continued refluxed overnight. Then, silica gel (about 2.00 g) was added to the reaction to hydrolyze bromide and the mixture was continued to refluxed for 2 h. The reaction mixture was purified by column chromatography (silica gel, dichloromethane/methanol = 100/1, *v*/*v*) to afford compound **3** (0.79 g, 0.27 mmol, 15%) as an orange solid. Since compound **3** is difficult to purify, we have improved the synthetic route to obtain the crude product by preliminary purification, which was directly used in the next step. Next, compound **3** (0.50 g, 0.17 mmol), pyridinium dichromate (PDC, 0.26 g, 0.68 mmol) and dichloromethane (4 mL) were added to a pressure-resistant reaction flask (15 mL) and the mixture was stirred and heated to reflux for 1.5 h. Then, the reaction mixture was filtered, concentrated and purified by column chromatography (silica gel, petroleum ether/dichloromethane = 1/1, *v/v*) to get compound **4** (0.31 g, 0.16 mmol, 97%) as an orange solid. ^1^H NMR (400 MHz, CDCl_3_, 298 K) δ 7.13 (s, 2H), 7.07 (s, 2H), 6.89 (s, 2H), 6.77 (s, 2H), 6.07 (s, 2H), 4.40–4.22 (m, 18H), 3.95 (s, 8H), 3.79–3.68 (m, 14H), 3.56 (t, *J* = 5.9 Hz, 4H), 3.12 (brs, 4H).


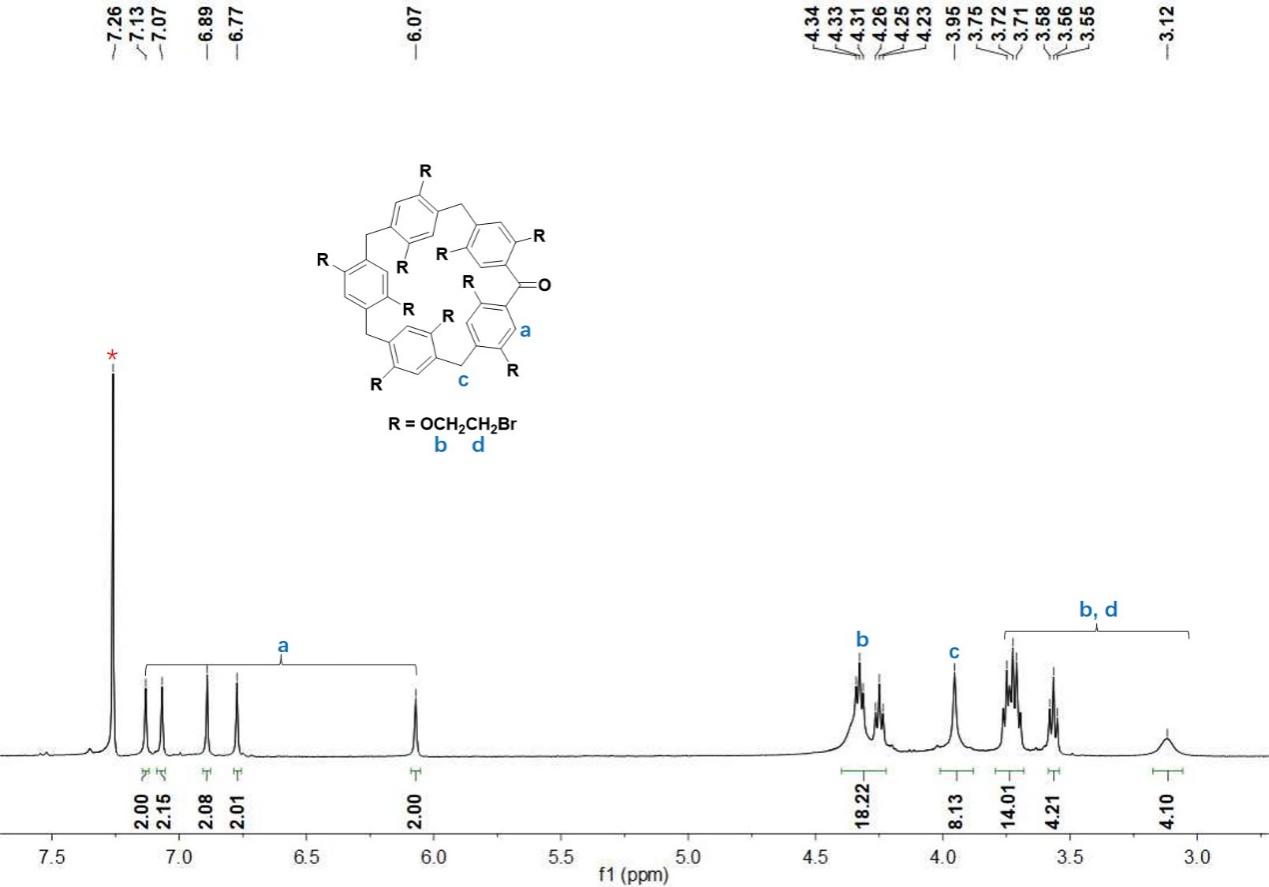


**Figure S3.** ^1^H NMR spectrum (400 MHz, CDCl_3_, 298 K) of compound **4** (* refers to solvent peak).

**Synthesis of compound** **5**^[S5]^

The synthesis of compound **5** was adapted from a previously reported method.^[S5]^ A solution of 1,4-dimethoxybenzene (10.0 g, 60.0 mmol) and trioxymethylene (1.86 g, 60.0 mmol) in dichloromethane (400 mL) was cooled to 0 ˚C using an ice bath for 10 min. Boron trifluoride diethyl etherate (5 mL) was then added dropwise, and the mixture was stirred at room temperature for 1.5 h. Then, water (200 mL) was added to quench the reaction. After suction filtration, separation and drying, the organic phase was concentrated under reduced pressure. The crude product was purified by silica gel chromatography (silica gel, petroleum ether/ dichloromethane = 2/1, *v*/*v*) to get compound **5** as a white solid (8.0 g, 8.99 mmol, 75%). ^1^H NMR (400 MHz, CDCl_3_, 298 K) δ 6.72 (s, 10H), 3.82 (q, *J* = 6.9 Hz, 20H), 3.77 (s, 10H), 1.26 (t, *J* = 6.9 Hz, 30H).


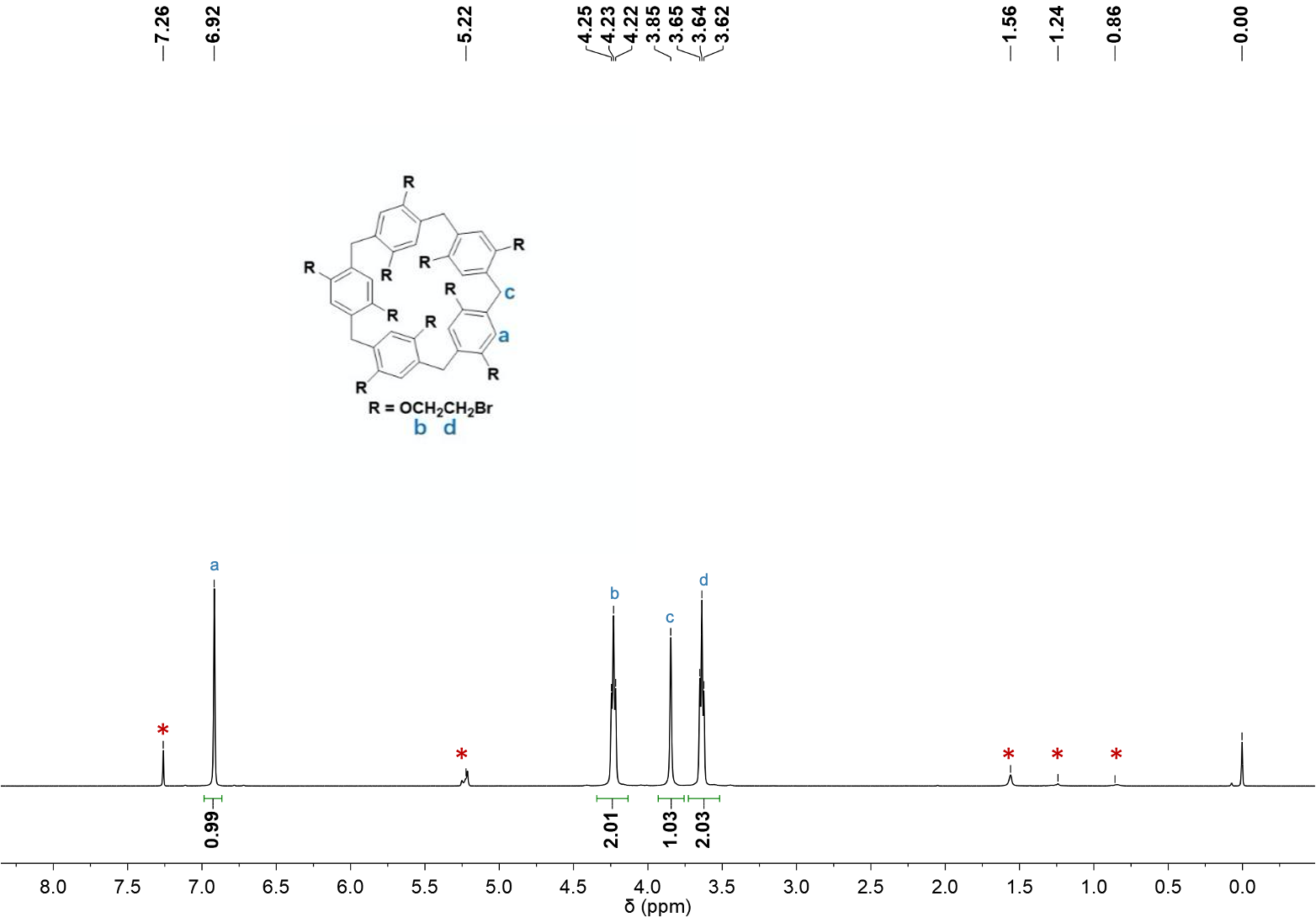


**Figure S4.** ^1^H NMR spectrum (400 MHz, CDCl_3_, 298 K) of compound **5** (* refers to solvent peak).

**Synthesis of compound** **6**^[S5]^

To a solution of compound **5** (2.00 g, 2.67 mmol) in chloroform (30 mL), boron tribromide (5.2 mL, 53.4 mmol) was added under under nitrogen atmosphere. The mixture was stirred at room temperature for 96 h. Then, 10 mL water was added to quench the reaction, and the resulting white precipitation was collected by vacuum filtration, washed with water (3 × 30 mL), and dried under vacuum to afford compound **6** as a white solid (1.34 g, 2.20 mmol, 98%). ^1^H NMR (400 MHz, DMSO-*d*_6_, 298 K) δ 8.43 (s, 10H), 6.57 (s, 10H), 3.42 (s, 10H).


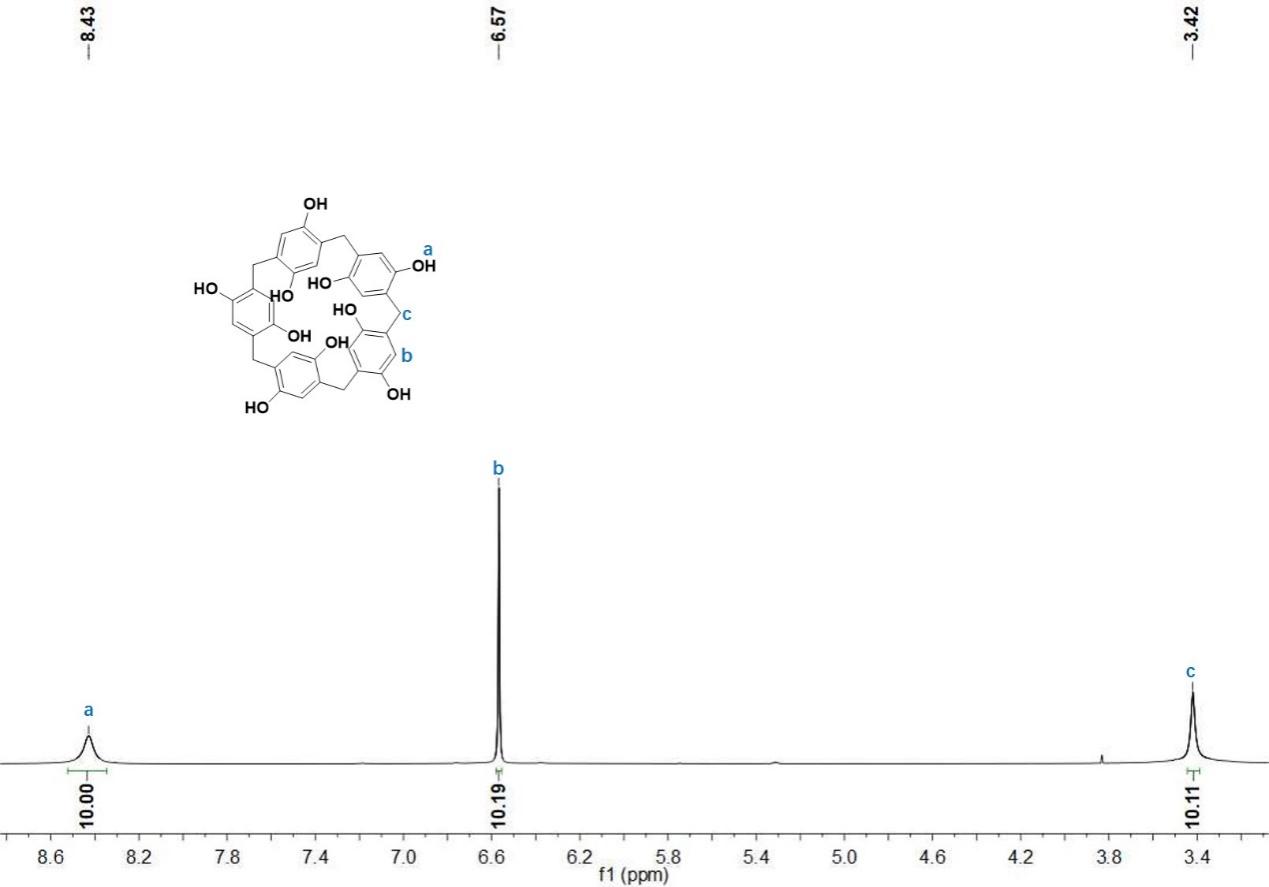


**Figure S5.** ^1^H NMR spectrum (400 MHz, DMSO-*d*_6_, 298 K) of compound **6**.

**Synthesis of compound** **7**^[S6]^

A mixture of compound **6** (2.69 g, 4.40 mmol) and K_2_CO_3_ (17.0 g, 0.12 mol) in dry CH_3_CN (200 mL) were added to a 500 mL three-necked flask and vigorously stirred under nitrogen atmosphere for 10 min. SO_2_F_2_ gas (sulfuryl fluoride) was introduced to the flask and the mixture was stirred at room temperature overnight. Then, the mixture was concentrated and purified by column chromatography (silica gel, petroleum ether/ethyl acetate = 6/1, *v/v*) to get compound **7** (3.30 g, 2.23 mmol, 51%) as a white solid. ^1^H NMR (400 MHz, DMSO-*d_6_*, 298 K) δ 7.68 (s, 10H), 4.26 (s, 10H).


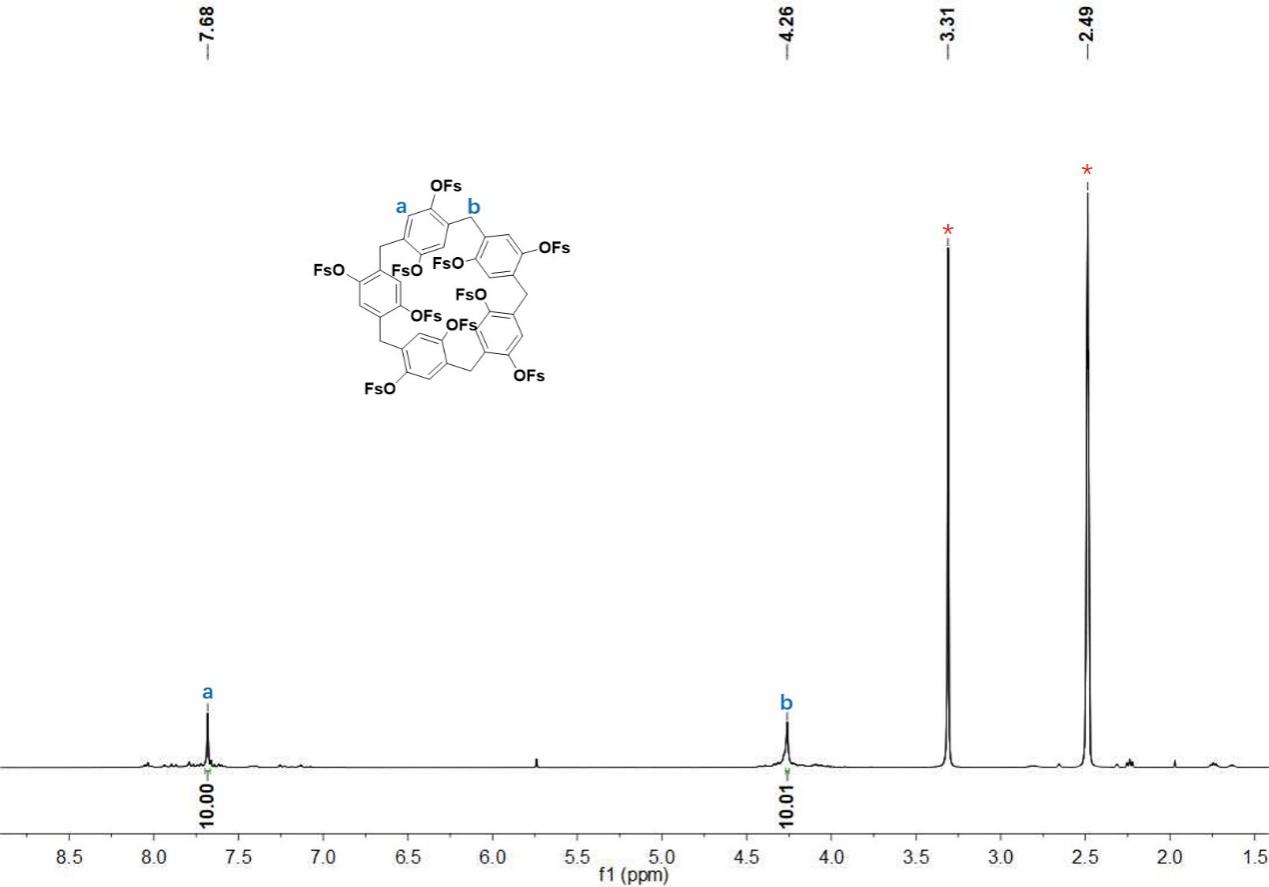


**Figure S6.** ^1^H NMR spectrum (400 MHz, DMSO-*d*_6_, 298 K) of compound **7** (* refers to solvent peak).

**Synthesis of compound** **8**^[S6]^

A mixture of compound **7** (0.73 g, 0.50 mmol), Pd(OAc)_2_ (0.90 g, 0.40 mmol), and 1,3-bis(diphenylphosphino)propane (0.24 g, 0.50 mmol) in DMSO (25 mL) were added to a 50 mL three-necked flask and stirred under a nitrogen atmosphere for 10 min. Et_3_N (3.44 mL, 25.0 mmol) was added to the mixture. After 10 min, HCOOH (0.93 mL, 25.0 mmol) was added to the reaction solution slowly. The mixture was stirred at room temperature overnight. Then, the solution was washed by water (3 × 50 mL) and the resulting organic phase was purified by column chromatography (silica gel, petroleum ether/ethyl acetate = 20/1, *v/v*) to give compound **8** (0.11 g, 0.24 mmol, 47%) as a white solid. ^1^H NMR (400 MHz, CDCl_3_, 298 K) δ 6.96 (s, 20H), 3.78 (s, 10H).


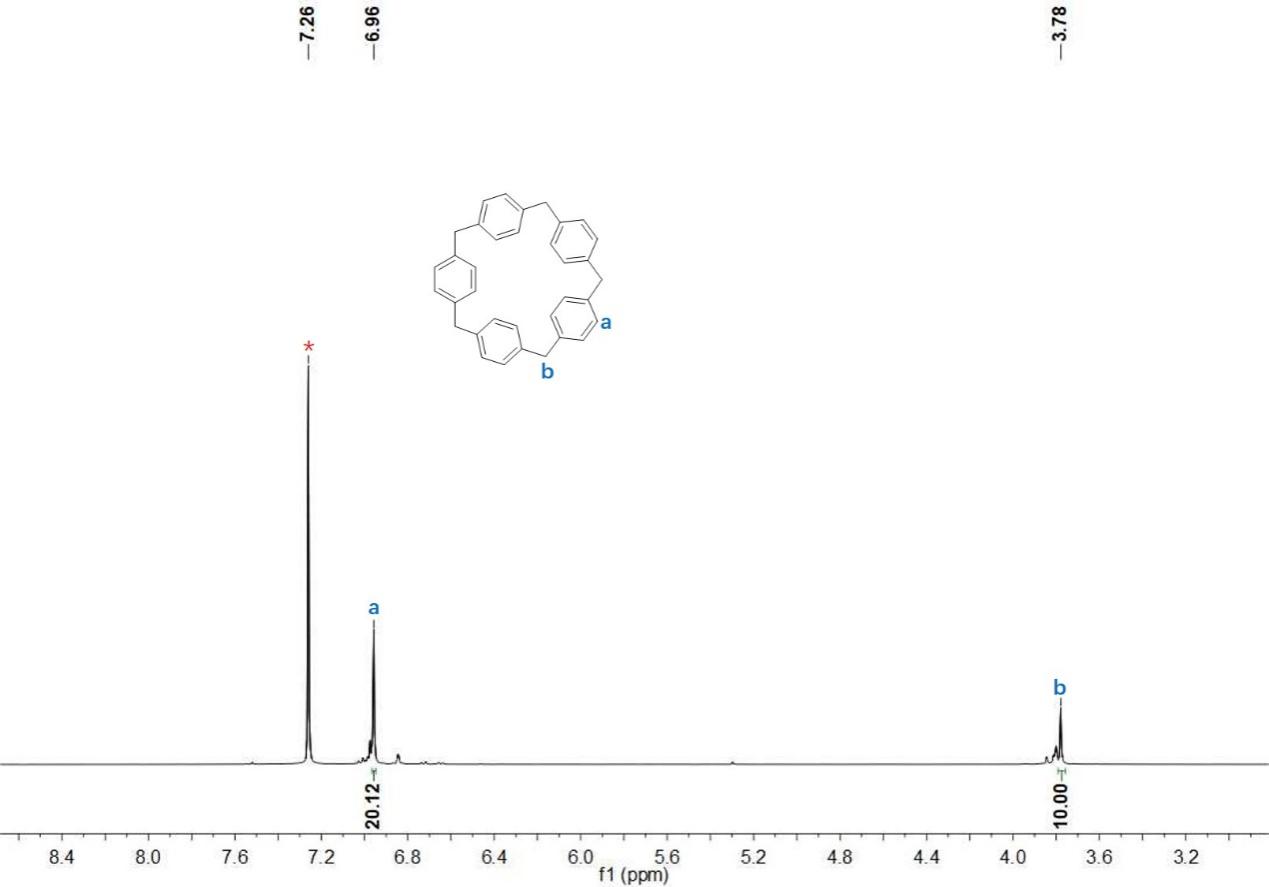


**Figure S7.** ^1^H NMR spectrum (400 MHz, CDCl_3_, 298 K) of compound **8** (* refers to solvent peak).

**Synthesis of compound** **9** **and 10**^[S7]^

Compound **8** (0.82 g, 1.81 mmol), azodiisobutyronitrile (AIBN, 0.02 g, 0.09 mmol), and *N*-bromosuccinimide (NBS, 0.39 g, 2.18 mmol) were dissolved in CCl_4_ (10 mL) and transferred to a 25 mL three-necked flask. The mixture was stirred, heated to reflux, and maintained under a nitrogen atmosphere. Among them, NBS was gradually added into the reaction mixture in batches over a period of 6 h. The mixture was refluxed overnight. Compound **8** (0.82 g, 1.81 mmol) and azodiisobutyronitrile (AIBN, 0.02 g, 0.09 mmol) were dissolved in CCl_4_ (5 mL) and then transferred to a 25 mL three-necked flask. The mixture was stirred and heated to reflux under a nitrogen atmosphere. And *N*-bromosuccinimide (NBS, 0.39 g, 2.18 mmol) dissolved in CCl_4_ (5 mL) was slowly added into the reaction mixture within 6 h. The mixture was continued refluxed overnight. Then, silica gel (about 2.00 g) was added to the reaction mixture to hydrolyze the brominated product, and the solution was refluxed for an additional 2 h. The reaction mixture was purified by column chromatography (silica gel, dichloromethane/methanol = 100/1, *v*/*v*) to give compound **9** as a yellow solid (0.22 g, 0.47 mmol, 26%). Since compound **9** is difficult to purify, we have improved the synthetic route to obtain the crude product by preliminary purification, which was directly used in the next step. Subsequently, compound **9** (0.22 g, 0.47 mmol), pyridinium dichromate (PDC, 0.53 g, 1.41 mmol), and dichloromethane (5 mL) were added to a pressure-resistant reaction flask (15 mL), and the mixture was stirred and heated under reflux for 2 h. After completion, the reaction mixture was filtered, concentrated, and purified by column chromatography (silica gel, petroleum ether/dichloromethane = 2/1, *v/v*), yielding compound **10** (0.21 g, 0.45 mmol, 95%) as a yellow solid. ^1^H NMR (400 MHz, CDCl_3_, 298 K) δ 7.24 (d, *J* = 8.3 Hz, 4H), 7.15 (d, *J* = 7.9 Hz, 4H), 7.05 (d, *J* = 7.9 Hz, 4H), 6.93 (s, 4H), 6.90 (d, *J* = 8.3 Hz, 4H), 3.96 (s, 4H), 3.88 (s, 4H).


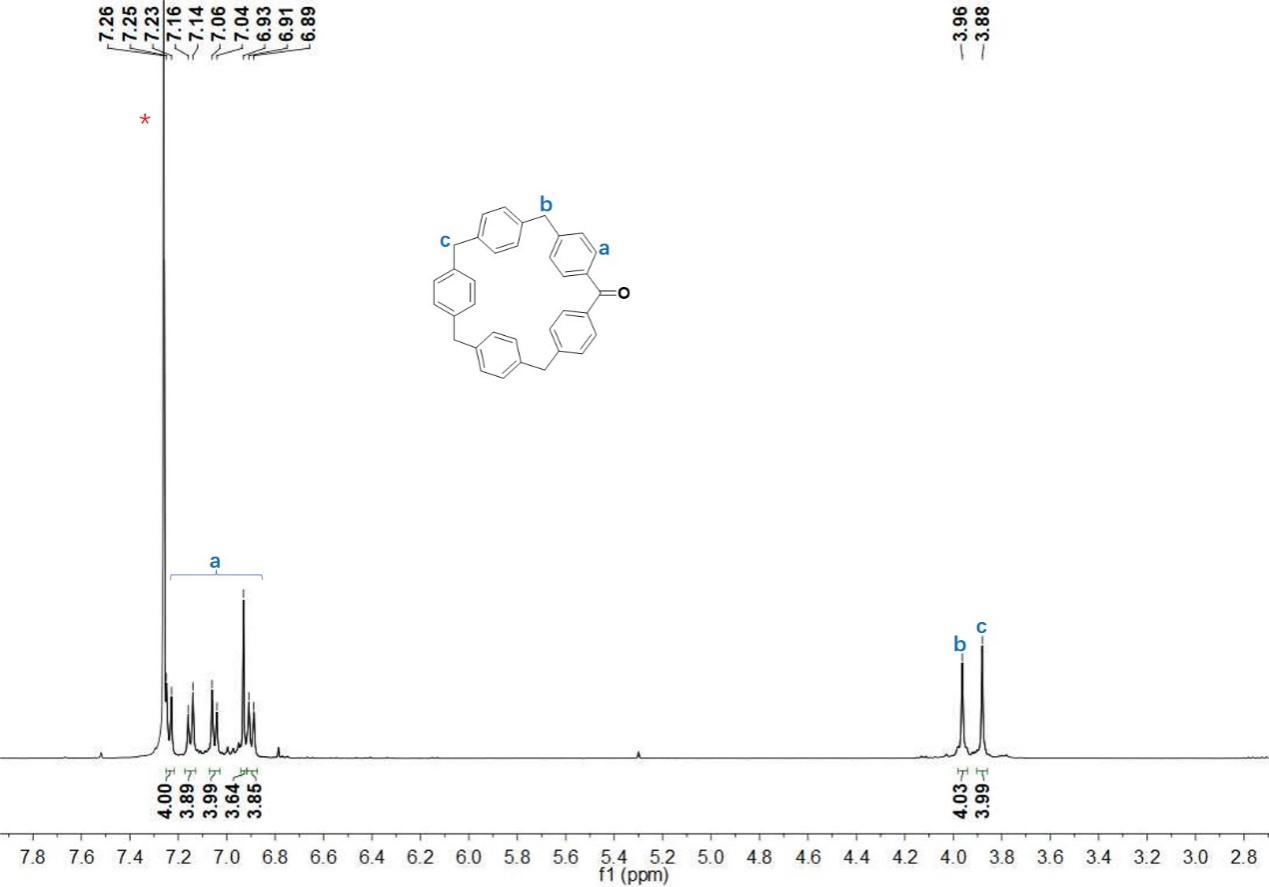


**Figure S8.** ^1^H NMR spectrum (400 MHz, CDCl_3_, 298 K) of compound **10** (* refers to solvent peak).

**Synthesis of compound** **11**

A 50 mL pressure-resistant bottle was charged with zinc powder (0.18 g, 2.71 mmol), compound **4** (0.12 g, 0.07 mmol), compound **10** (0.22 g, 0.47 mmol), and anhydrous tetrahydrofuran (6 mL) under a nitrogen atmosphere. The mixture was cooled to 0 ˚C and stirred for 30 mins. Then, TiCl_4_ (0.24 mL, 1.68 mmol) was added dropwise. After stirring for 1 h, the solution was heated under reflux overnight. The reaction was then quenched by saturated NaHCO_3_ solution (10 mL). After filtration, the filtrate was extracted with dichloromethane. The organic phase was dried over anhydrous sodium sulfate, concentrated, and purified by column chromatography (silica gel, petroleum ether/dichloromethane = 2/1, *v*/*v*) to give compound **11** as a white solid (0.044 g, 0.021 mmol, 33%). ^1^HNMR (400 MHz, CDCl_3_, 298 K) δ 7.04–6.91 (m, 20H), 6.75 (m, 6H), 6.54 (s, 2H), 6.39 (s, 2H), 4.24 (m, 12H), 3.72–3.97 (m, 28H), 3.44 (m, 4H), 3.28 (m, 4H), 2.86 (m, 4H), 2.19 (m, 2H), 2.02 (m, 2H). ^13^C NMR (100 MHz, CDCl_3_, 298 K) δ 150.20, 149.79, 149.74, 149.65, 148.53, 142.45, 140.70, 139.61, 139.53, 139.30, 139.21, 130.64, 130.27, 130.09, 129.37, 129.22, 129.02, 128.60, 128.40, 127.65, 118.48, 116.19, 116.08, 115.94, 115.84, 69.04, 68.91, 68.45, 41.40, 41.22, 30.73, 30.69, 30.45, 30.26, 29.86, 29.70, 29.53.


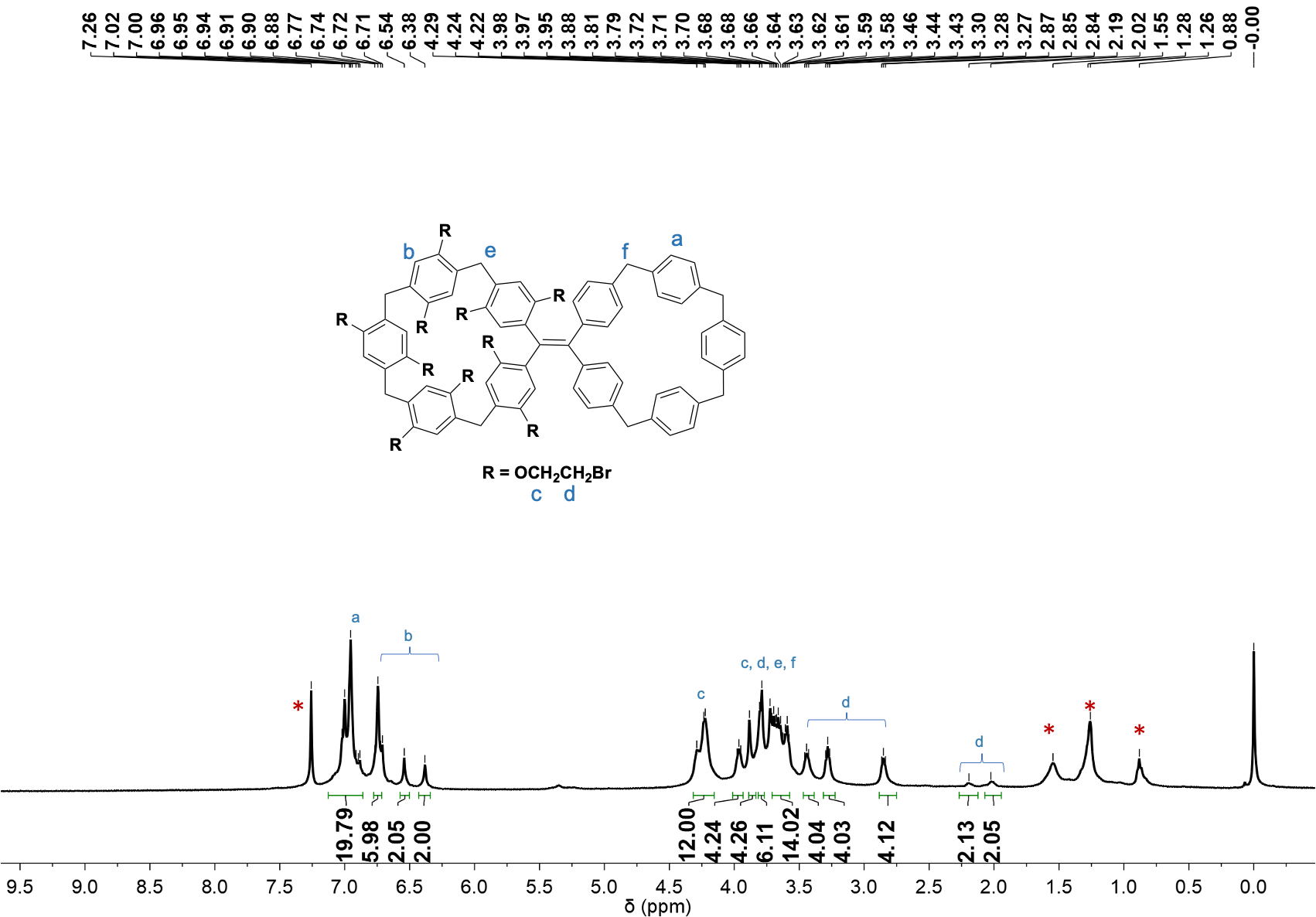


**Figure S9.** ^1^H NMR spectrum (400 MHz, CDCl_3_, 358 K) of compound **11** (* refers to solvent peak).


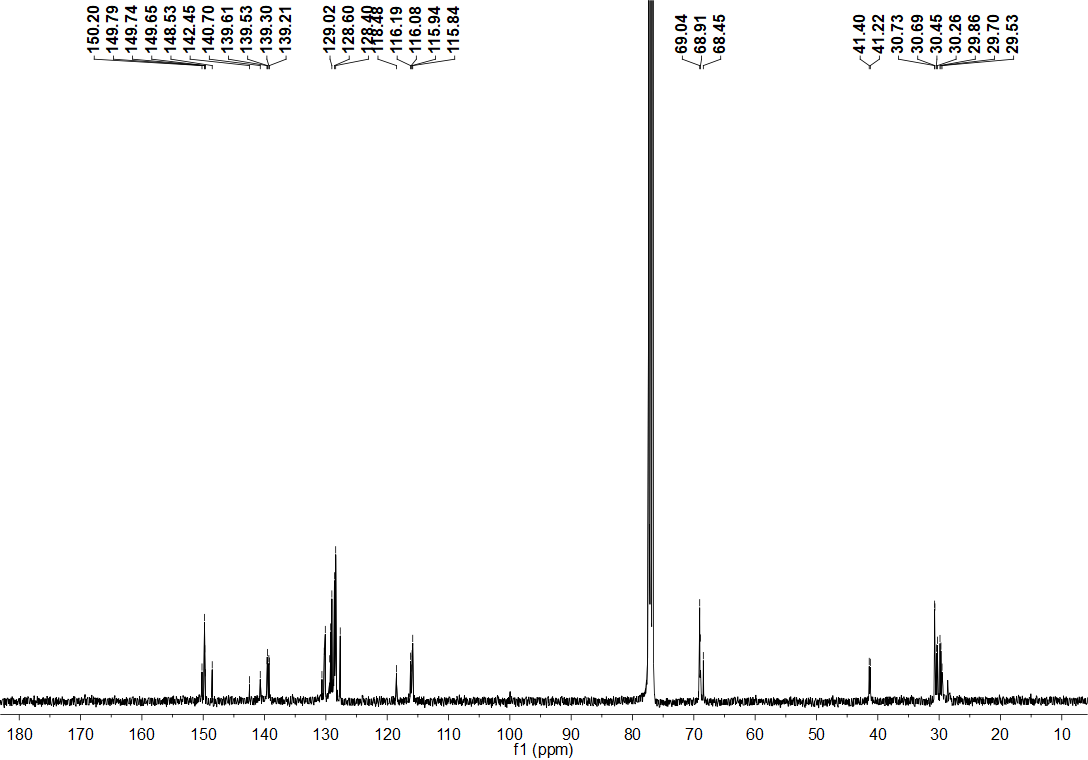


**Figure S10.** ^13^C NMR spectrum (100 MHz, CDCl_3_, 298 K) of compound **11** (* refers to solvent peak).

**Synthesis of compound** ***m*-TPE WP5-PCP**

To a solution of **11** (0.030 g, 0.01 mmol) in THF (1 mL), trimethylamine (33% in ethanol, 1 mL, 3.70 mmol) was added. The mixture was stirred and refluxed for three days. Then, the solvent was removed under vacuum to give compound ***m*-TPE WP5-PCP** as a white solid (0.036 g, 0.013 mmol, 96%). Note: Although the precursor compound **11** was treated with excess trimethylamine, steric hindrance around the peripheral reactive sites might prevent the complete quaternization of all side-chains. Therefore, the isolated material is more conservatively described as a highly quaternized product containing closely related species with different extents of quaternization, rather than an unambiguously identified single decacationic compound.


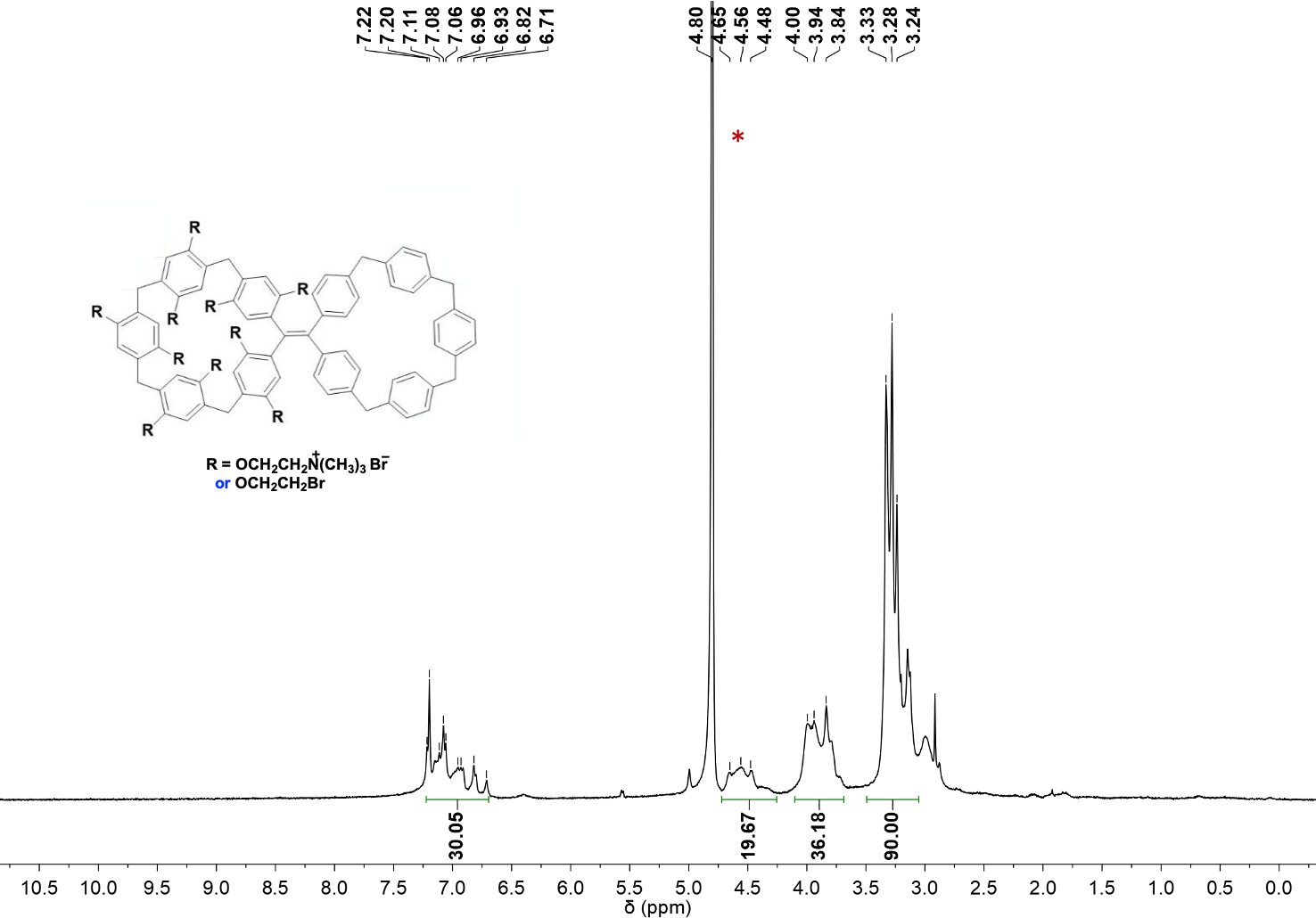


**Figure S11.** ^1^H NMR spectrum (400 MHz, D_2_O, 298 K) of compound ***m*-TPE WP5-PCP** (* refers to solvent peak).


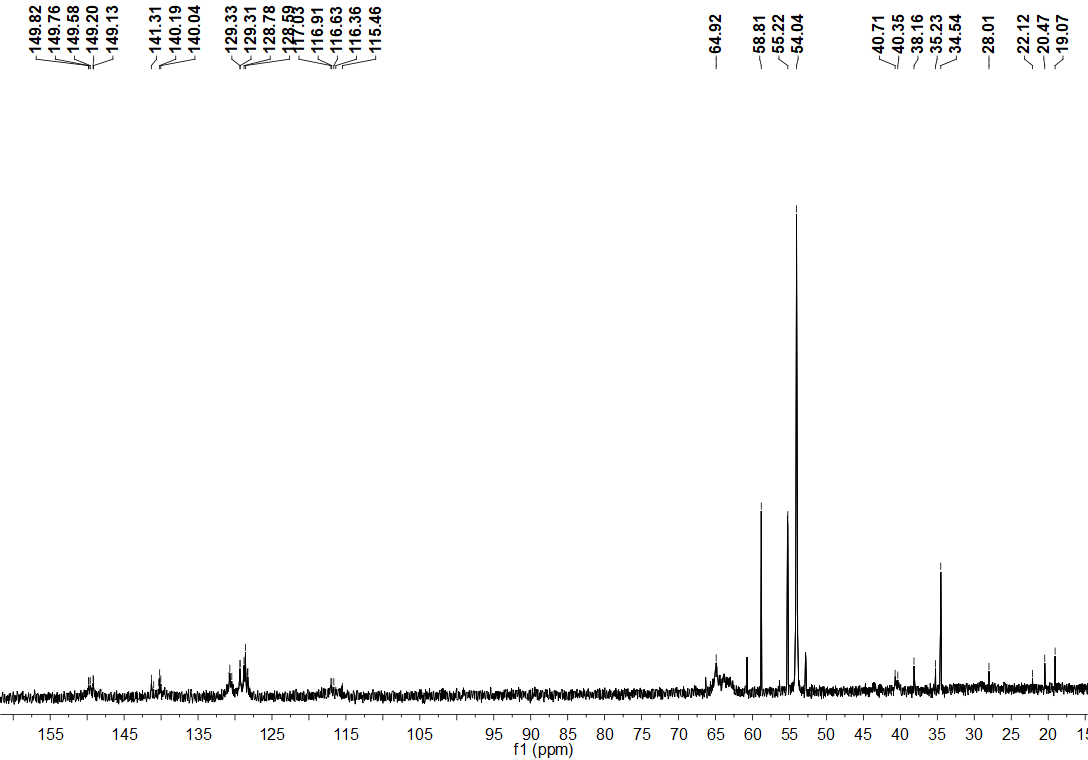


**Figure S12.** ^13^C NMR spectrum (100 MHz, D_2_O, 298 K) of compound ***m*-TPE WP5-PCP** (* refers to solvent peak).

**Synthesis of guest molecule**

**Scheme S2.** Synthesis route of guest molecule.

**Synthesis of compound** **12**

To a solution of 4-nitrobenzoic acid (10.0 g, 59.8 mmol) in deionized water (150 mL) was added NaOH (33.4 g, 833 mmol), and the mixture was heated up to 70 ˚C. Then, D-glucose (66.7 g, 370 mmol) in deionized water (50 mL) was slowly added, and the reaction mixture was stirred overnight. After filtration, the precipitation was dissolved in water, and acetic acid (20 mL) was added, followed by filtration to afford compound **12** as ab orange solid (4.20 g, 14.6 mmol, 49%). ^1^H NMR (400 MHz, DMSO-*d*_6_, 298 K) δ 13.07 (s, 2H), 8.16 (d, *J* = 8.3 Hz, 4H), 8.00 (d, *J* = 8.4 Hz, 4H).


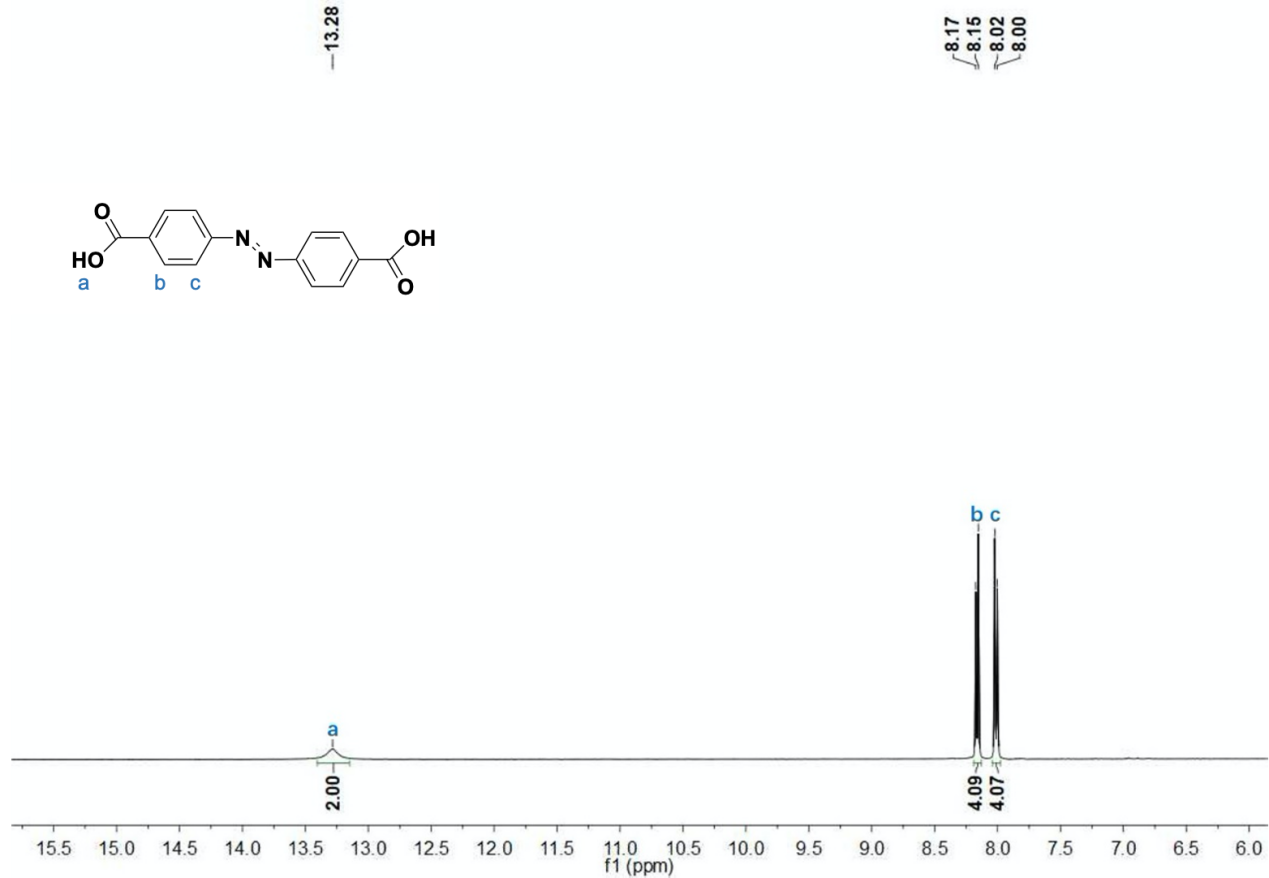


**Figure S13.** ^1^H NMR spectrum (400 MHz, DMSO-*d*_6_, 298 K) of compound **12**.

**Synthesis of Phe-G (L** or **D)**

To a suspension of compound **12** (0.34 g, 1.27 mmol) in dichloromethane (40 mL), L or D-phenylalanine methyl ester hydrochloride (0.55 g, 2.55 mmol) and 2-(7-Azabenzotriazol-1-yl)-*N*,*N*,*N'*,*N'*-tetramethyluronium hexafluorophosphate (HATU, 1.06 g, 2.78 mmol) were added. After stirring for 10 min, *N*,*N*-Diisopropylethylamine (1.2 mL) was added at −20 ˚C, and the reaction mixture was stirred at room temperature overnight. The resulting mixture was washed sequentially with 1 M aqueous HCl (3 × 5 mL), aqueous NaHCO_3_ (3 × 5 mL), and brine (3 × 5 mL), dried over NaSO_4_, and the solvent was removed under vacuum. To a suspension of the obtained product in THF (10 mL), 2 M aqueous NaOH (15 mL) was added slowly, and the mixture was stirred at room temperature for 4 h. The reaction mixture was then acidified with HCl. The precipitate was collected by filtration, washed with water, and dried under vacuum. Subsequently, the crude product (0.23 g, 0.55 mmol) and NaOH (0.02 g, 0.55 mmol) were dissolved in H_2_O (30 mL) and stirred at room temperature for 12 h. Water was removed under reduced pressure to give **Phe-G (L** or **D)**.

**Phe-G (D)** was obtained as an orange solid (0.59 g, 0.98 mmol, 78%). ^1^H NMR (400 MHz, DMSO-*d*_6_, 298 K) δ 8.05 (d, *J* = 7.0 Hz, 2H), 7.91 (s, 8H), 7.15–7.20 (m, 8H), 7.07–7.11 (m, 2H), 4.25–4.30 (m, 2H), 3.22–3.26 (m, 2H), 3.04–3.09 (m, 2H). ^13^C NMR (100 MHz, DMSO-*d*_6_, 298 K) δ 173.08, 164.55, 153.53, 140.09, 138.29, 129.94, 128.55, 128.12, 126.04, 123.13, 56.56, 37.77. HR-ESI-MS: m/z calcd. [M-2Na+H] ^−^: 563.1930, found: 563.1893.


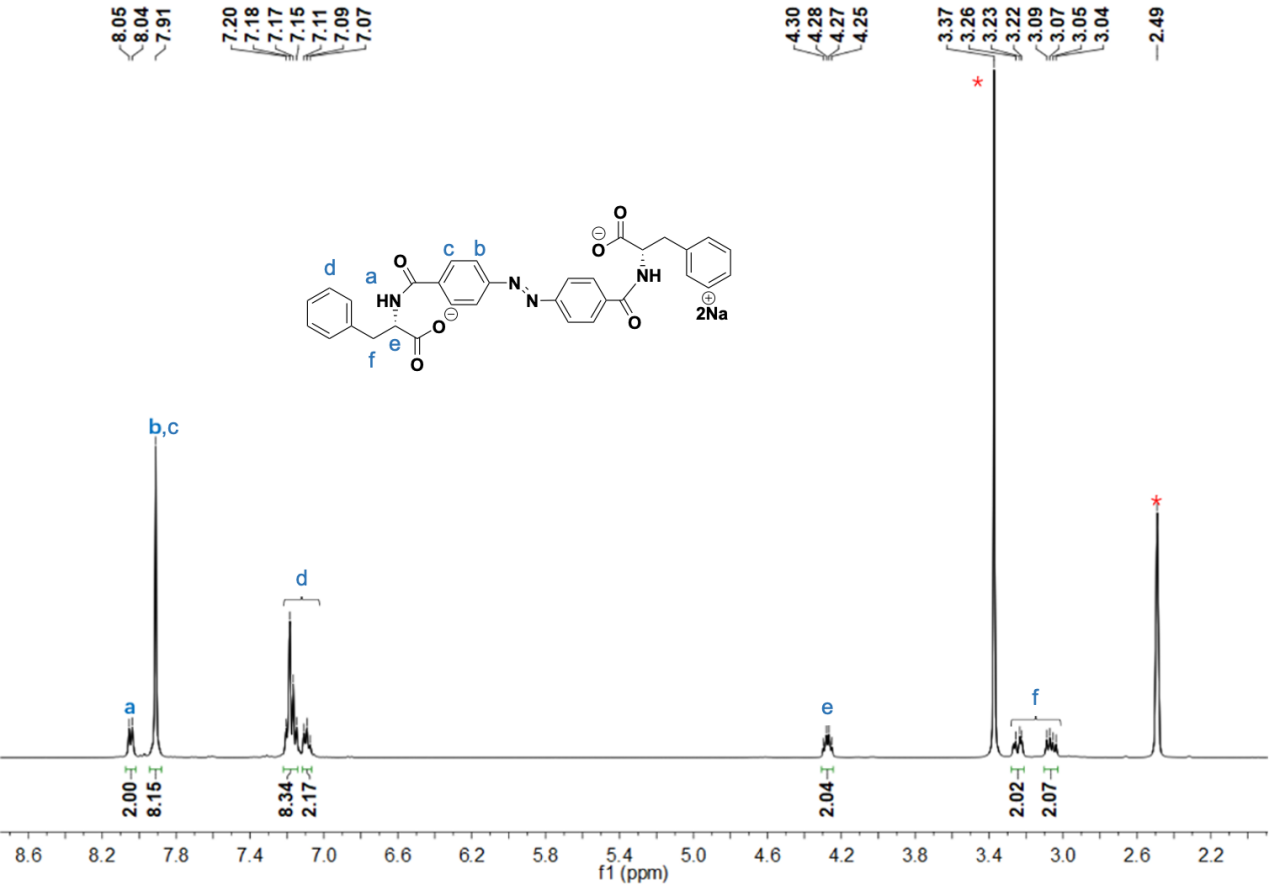


**Figure S14.** ^1^H NMR spectrum (400 MHz, DMSO-*d*_6_, 298 K) of **Phe-G (D)**.


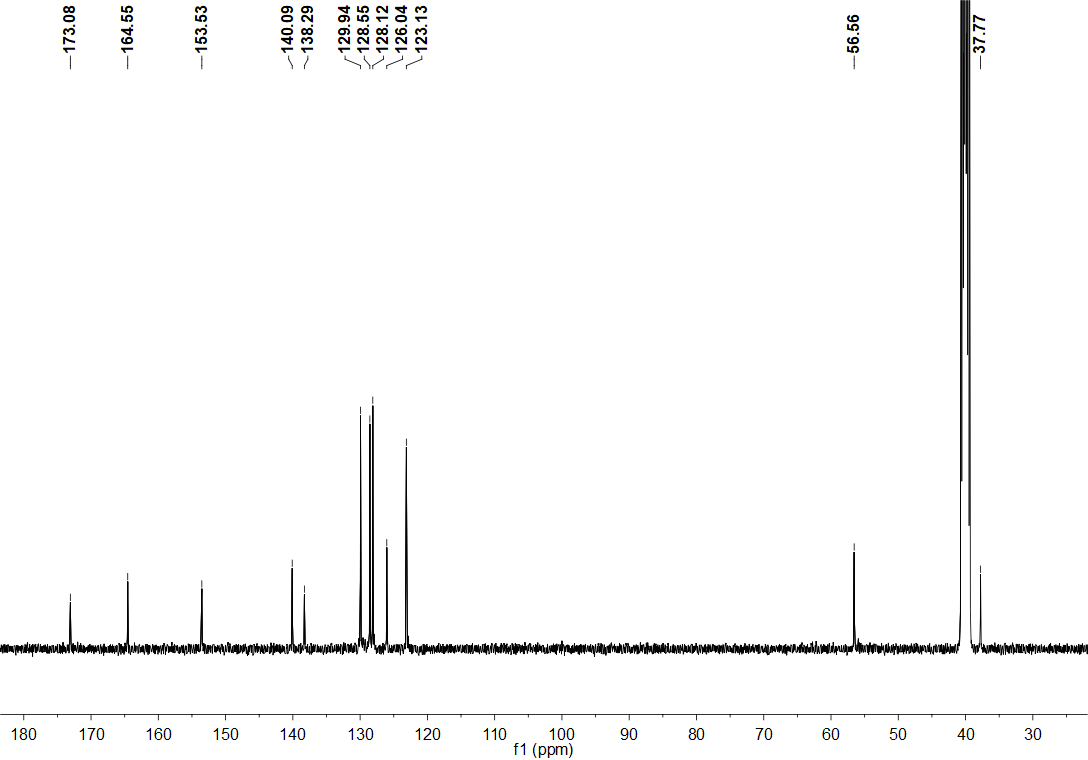


**Figure S15.** ^13^C NMR spectrum (100 MHz, DMSO-*d*_6_, 298 K) of **Phe-G (D)**.


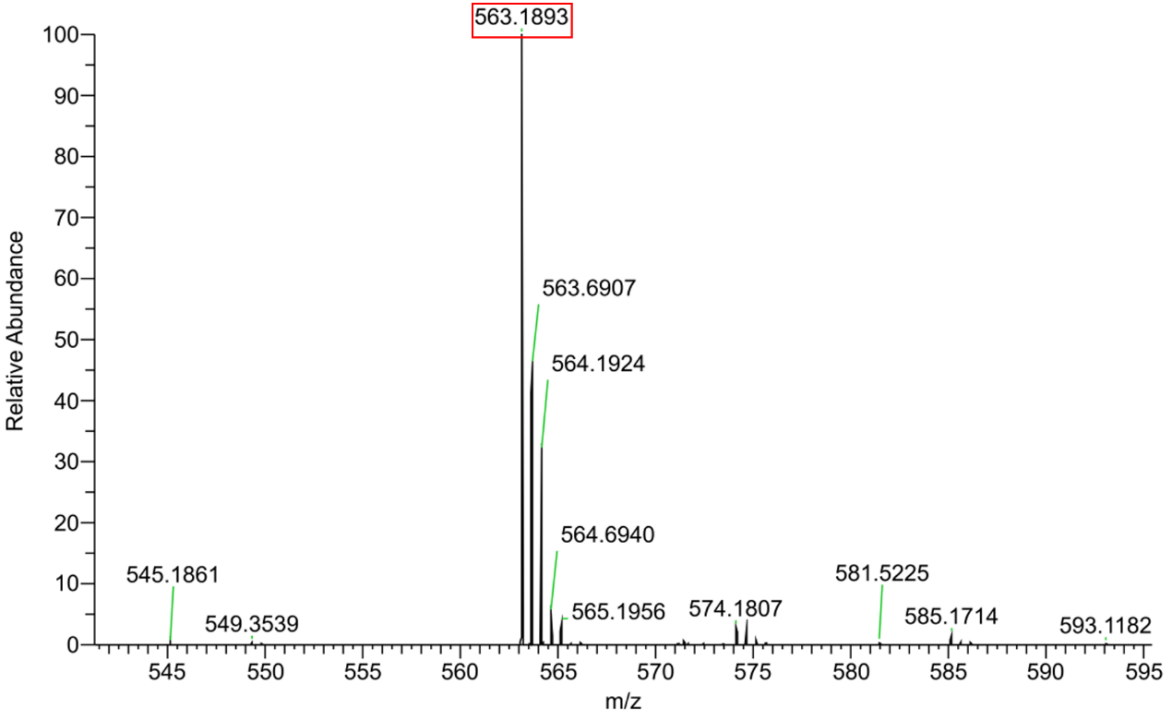


**Figure S16.** HR-ESI-MS spectrum of **Phe-G (D)**.

**Phe-G (L)** was obtained as an orange solid (0.60 g, 1.00 mmol, 80%).^1^H NMR (400 MHz, DMSO-*d*_6_, 298 K) δ 8.00 (d, *J* = 7.0 Hz, 2H), 7.90–7.95 (m, 8H), 7.16–7.18 (m, 8H), 7.08–7.12 (m, 2H), 4.18–4.22 (m, 2H), 3.21–3.35 (m, 2H), 3.04–3.09 (m, 2H).


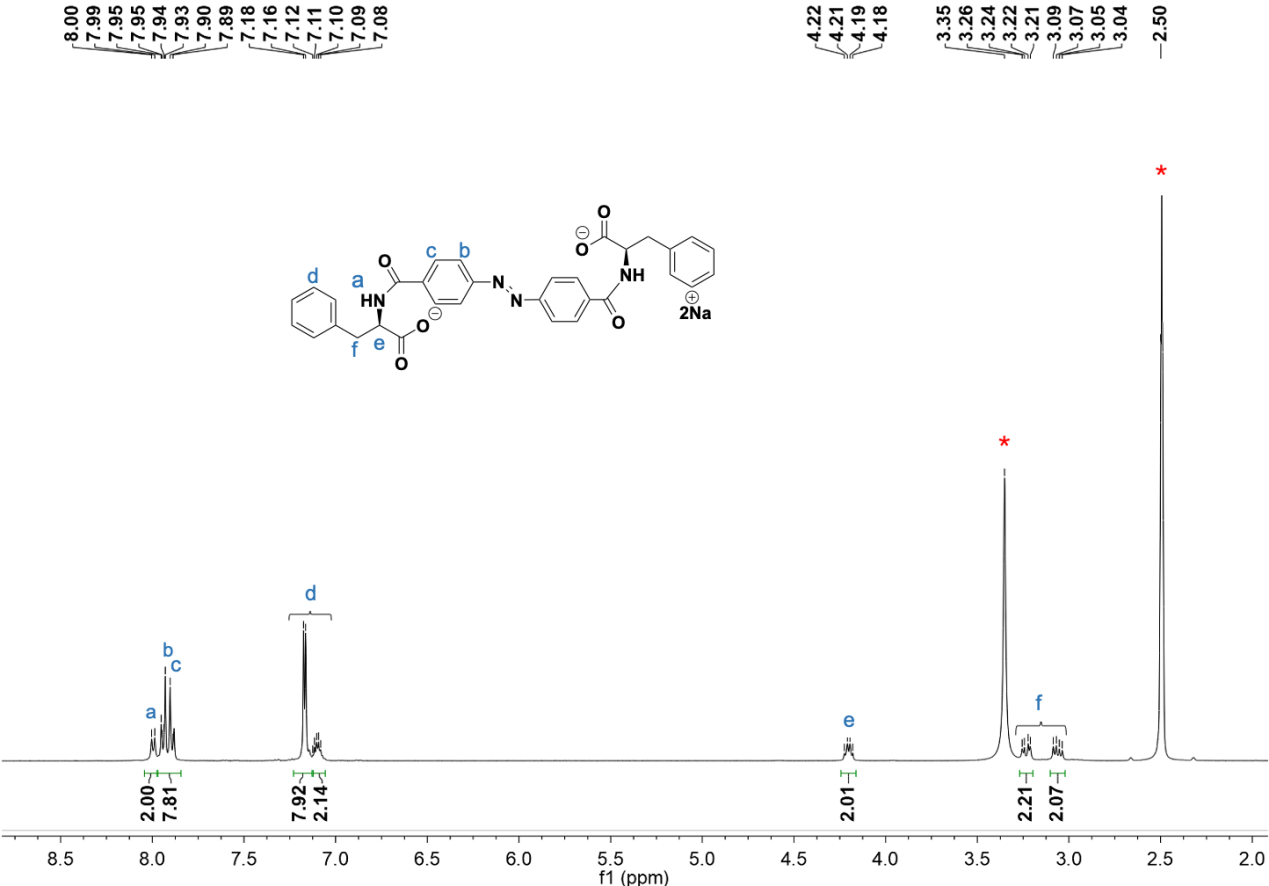


**Figure S17.** ^1^H NMR spectrum (400 MHz, DMSO-*d*_6_, 298 K) of **Phe-G (L)**.

**Synthesis of Ala-G (L** or **D)**

**Ala-G (D)** was obtained by replacing D-phenylalanine methyl ester hydrochloride with D-alanine ethyl ester hydrochloride to give an orange solid (0.25 g, 0.55 mmol, 44%). ^1^H NMR (400 MHz, DMSO-*d*_6_, 298 K) δ 8.20 (d, *J* = 6.3 Hz, 2H), 8.01 (d, *J* = 8.5 Hz, 4H), 7.94 (d, *J* = 8.4 Hz, 4H), 3.99–4.06 (m, 2H), 1.31 (d, *J* = 6.9 Hz, 6H). ^13^C NMR (100 MHz, DMSO-*d*_6_, 298 K) δ 174.54, 164.16, 153.56, 138.08, 128.59, 123.19, 50.96, 19.60. HR-ESI-MS: m/z calcd. [M-2Na+H]^−^: 411.1304, found: 411.1276.


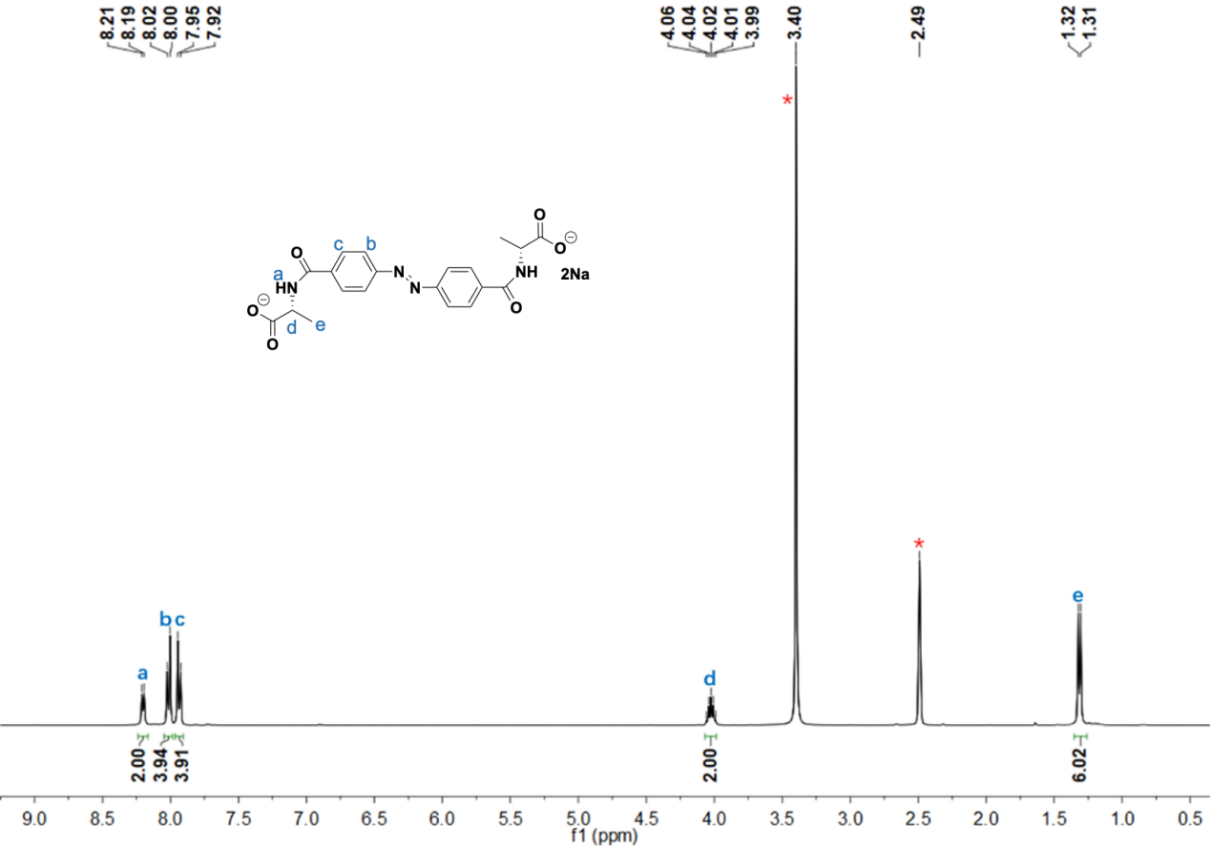


**Figure S18.** ^1^H NMR spectrum (400 MHz, DMSO-*d*_6_, 298 K) of **Ala-G (D)**.


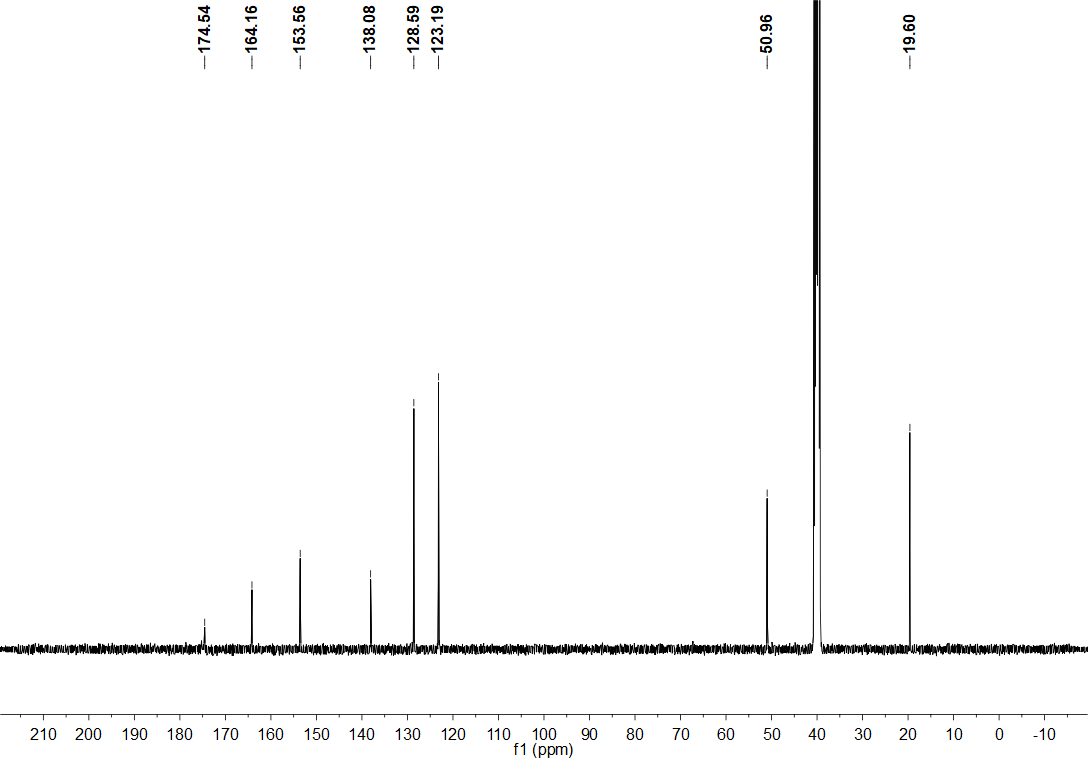


**Figure S19.** ^13^C NMR spectrum (100 MHz, DMSO-*d*_6_, 298 K) of **Ala-G (D)**.


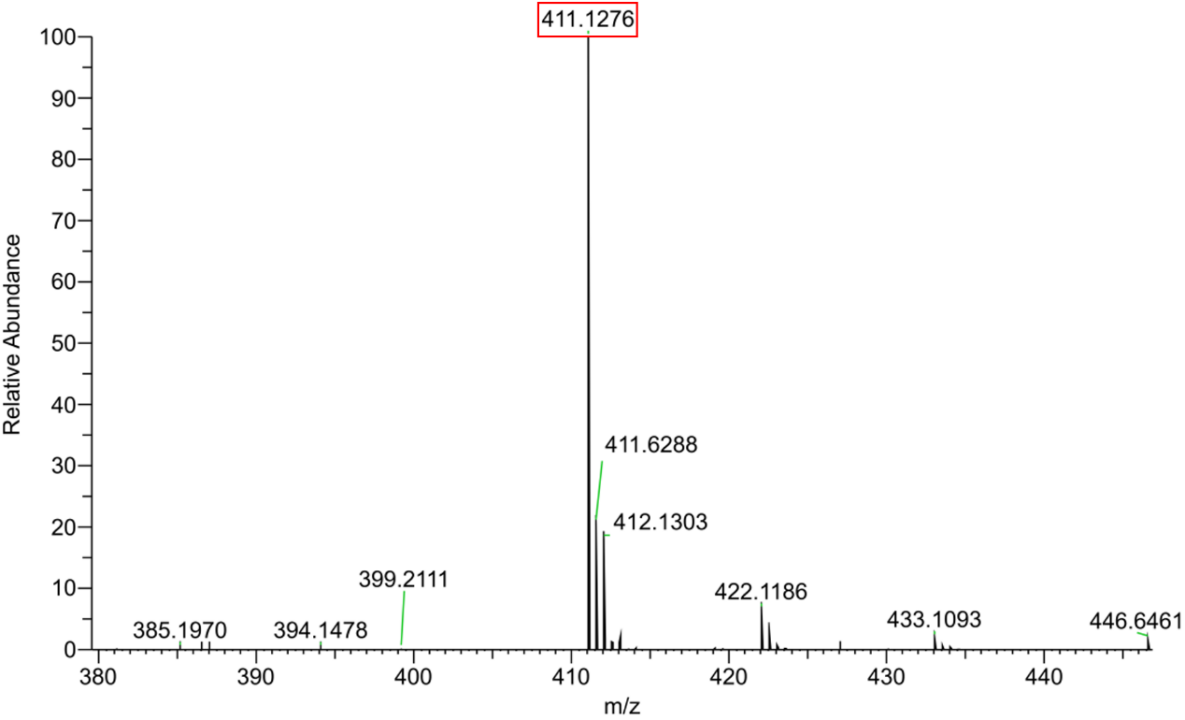


**Figure S20.** HR-ESI-MS spectrum of **Ala-G (D)**.

**Phe-G (L)** was obtained as an orange solid (0.25 g, 0.58 mmol, 46%). ^1^H NMR (400 MHz, DMSO-*d*_6_, 298 K) δ 8.23 (d, *J* = 6.4 Hz, 2H), 8.01 (d, *J* = 8.8 Hz, 4H), 7.98 (d, *J* = 8.8 Hz, 4H), 3.92–3.96 (m, 2H), 1.30 (d, *J* = 6.8 Hz, 6H).


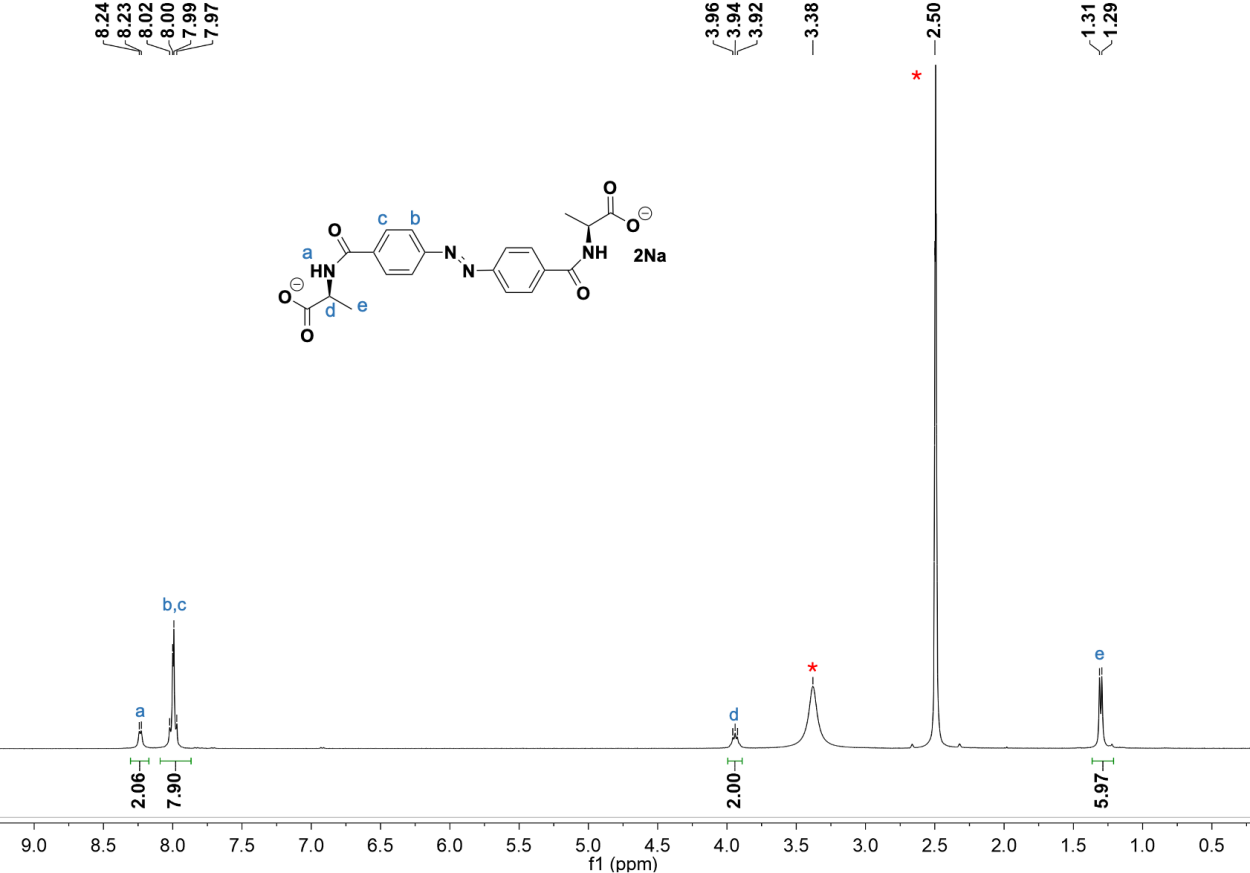


**Figure S21.** ^1^H NMR spectrum (400 MHz, DMSO-*d*_6_, 298 K) of **Ala-G (L)**.

1. **Supplementary Discussion**

**Single crystal structures of *m*-TPE WP5-PCP**


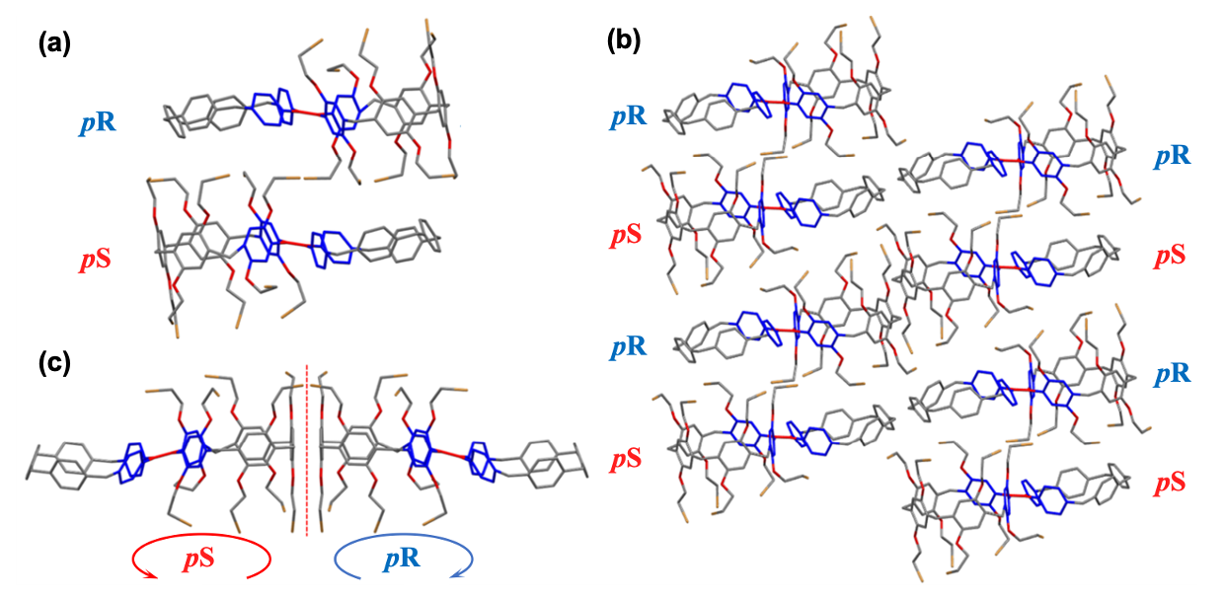


**Figure S22.** (a) Single crystal structures of *p*R-***m*-TPE WP5-PCP** and *p*S-***m*-TPE WP5-PCP** obtained from crystals. (b) Molecular packing form of ***m*-TPE WP5-PCP**. (c) Crystal structures of diastereomers of ***m*-TPE WP5-PCP**.

**Table S1.** X-ray crystallography data.

| Empirical formula | C_90_H_86_Br_10_O_10_ |
| --- | --- |
| Formula weight | 2126.59 |
| Temperature | 150 K |
| Wavelength | 1.54178 Å |
| Crystal system | triclinic |
| Space group | *P*-1 (2) |
| Unit cell dimensions | *a* = 12.3336(3) Å *α* =106.360(1)°.  *b* = 19.8623(5) Å *β* = 93.768(1)°.  *c* = 20.9033(5) Å *γ* = 94.870(1)°. |
| Volume | 4874.12(20) Å^3^ |
| *Z* | 2 |
| Density (calculated) | 1.449 Mg/m^3^ |
| Absorption coefficient | 5.301 mm^-1^ |
| *F*(000) | 2112 |
| Theta range for data collection | 2.212 to 72.560° |
| Index ranges | -15<=*h*<=15, -24<=*k*<=24, -25<=*l*<=25 |
| Reflections collected | 58613 |
| Independent reflections | 19173 [*R*(int) = 0.0408] |
| Completeness to theta = 67.679° | 99.7% |
| Refinement method | Full-matrix least-squares on *F*^2^ |
| Data / restraints / parameters | 19173 / 814 / 1259 |
| Goodness-of-fit on *F*^2^ | 1.996 |
| Final *R* indices [*I*>2sigma(*I*)] | *R*_1_ = 0.1459, w*R*_2_ = 0.4232 |
| *R* indices (all data) | *R*_1_ = 0.1643, w*R*_2_ = 0.4525 |

**Host/guest interaction of *m*-TPE WP5-PCP**


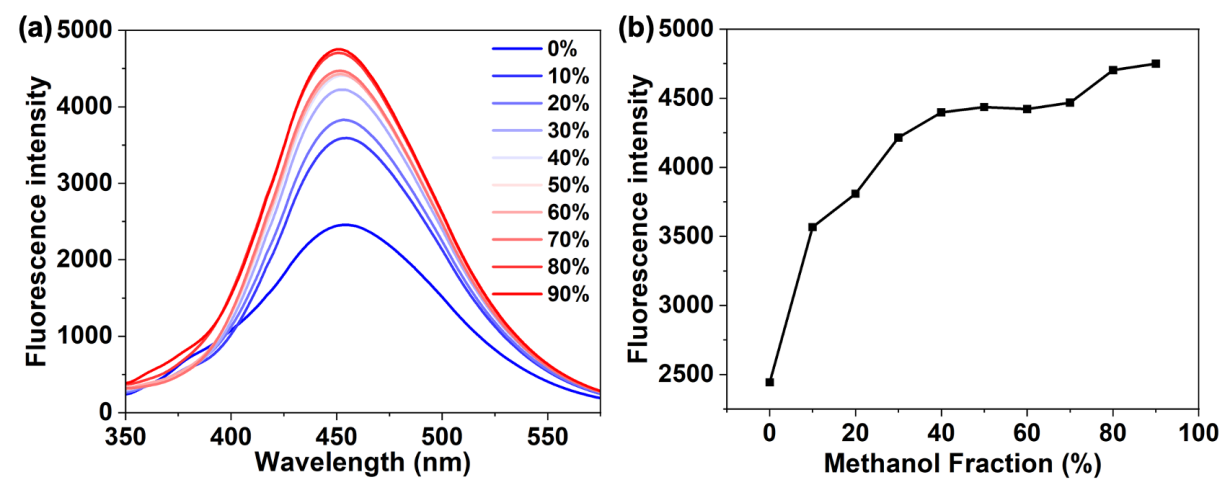


**Figure S23.** (a) Fluorescence spectra of ***m*-TPE WP5-PCP** (1 × 10^-5^ M) in THF/water mixture with different THF fractions upon UV light irradiation (300 nm). (b) Plot of the fluorescence intensity of ***m*-TPE WP5-PCP** (1 × 10^-5^ M) in THF/water mixture with different THF fractions.


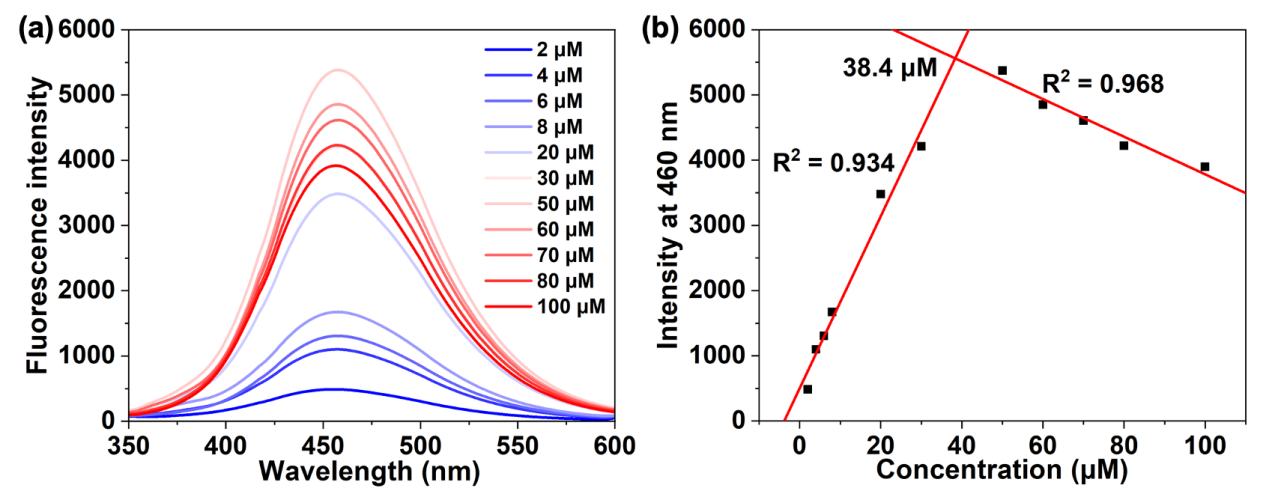


**Figure S24.** (a) The concentration-dependent fluorescence spectra of ***m*-TPE WP5-PCP** in water. (b) Dependence of the fluorescence intensity of ***m*-TPE WP5-PCP** in water under different concentrations at 460 nm.


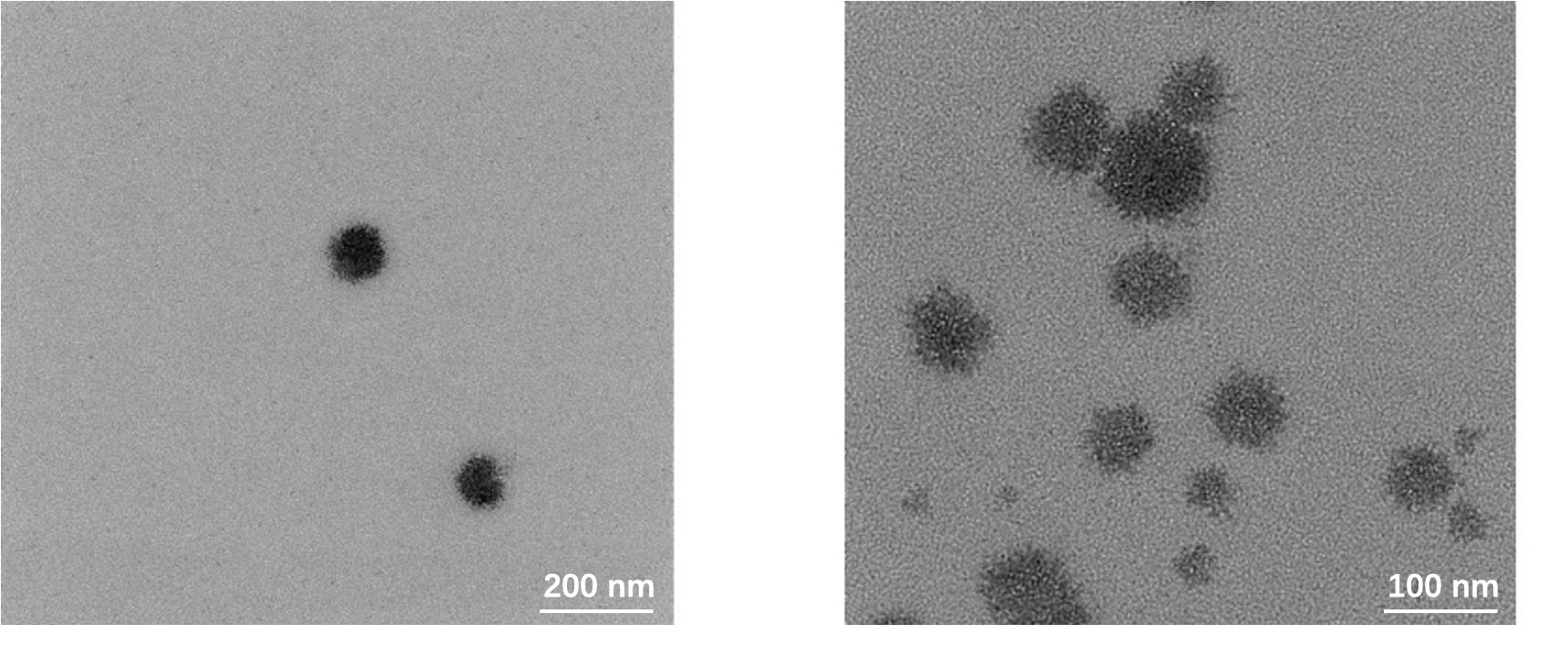


**Figure S25.** TEM images of ***m*-TPE WP5-PCP** (5.0 × 10^−4^ M) in water.


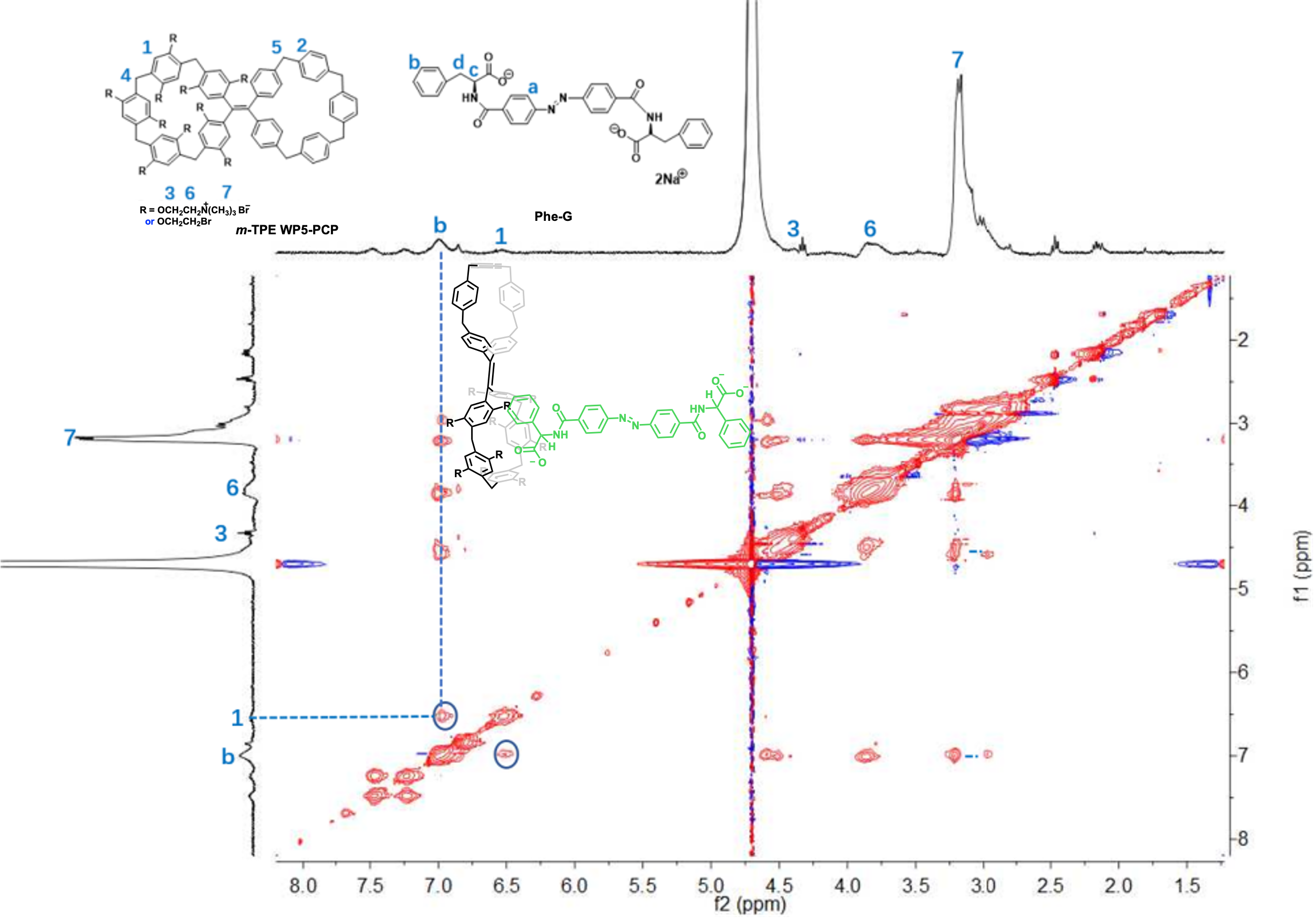


**Figure S26.** NOESY spectra of ***m*-TPE WP5-PCP** and **Phe-G** (D_2_O, 400 MHz, 298 K).


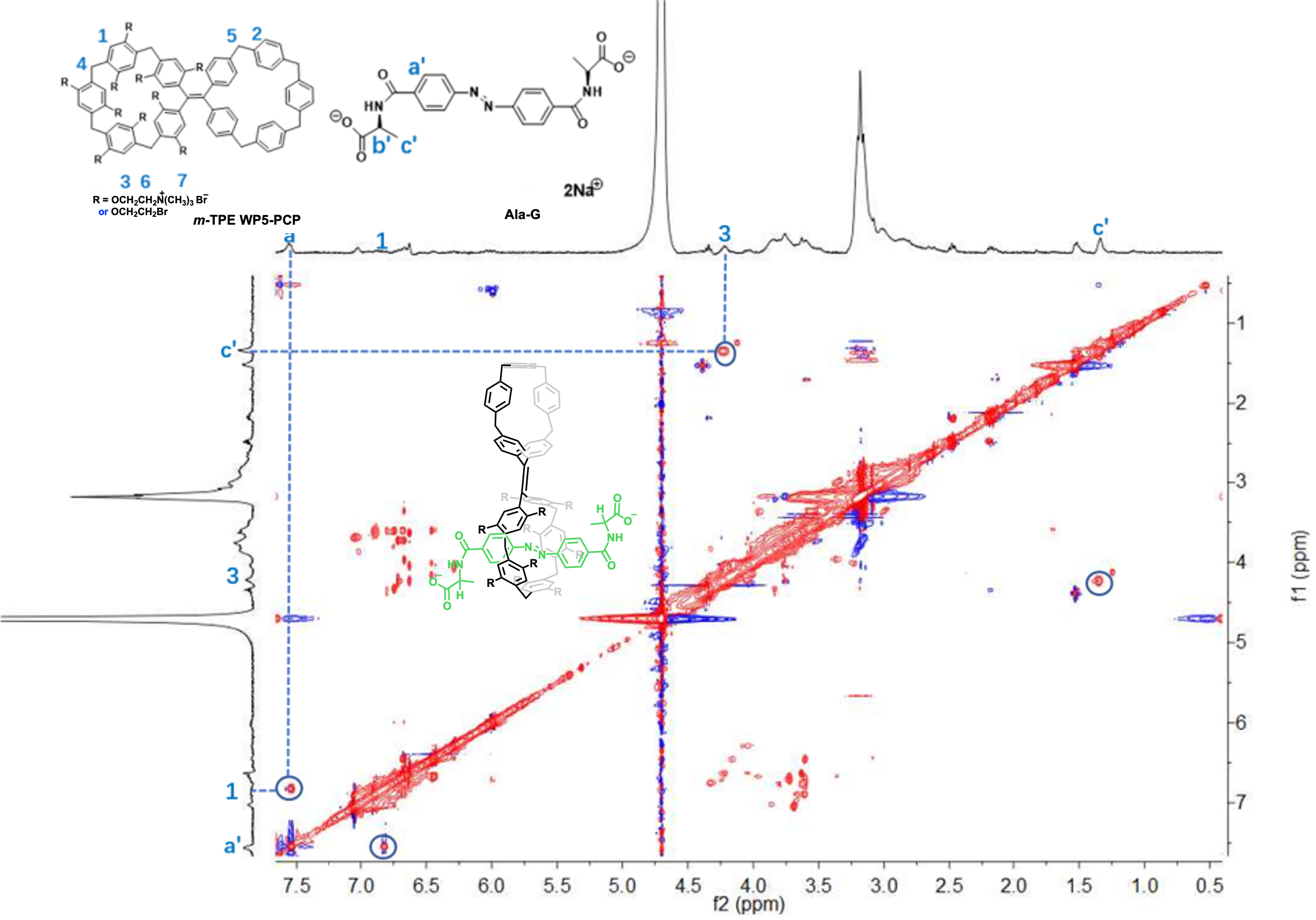


**Figure S27.** NOESY spectra of ***m*-TPE WP5-PCP** and **Ala-G** (D_2_O, 400 MHz, 298 K).


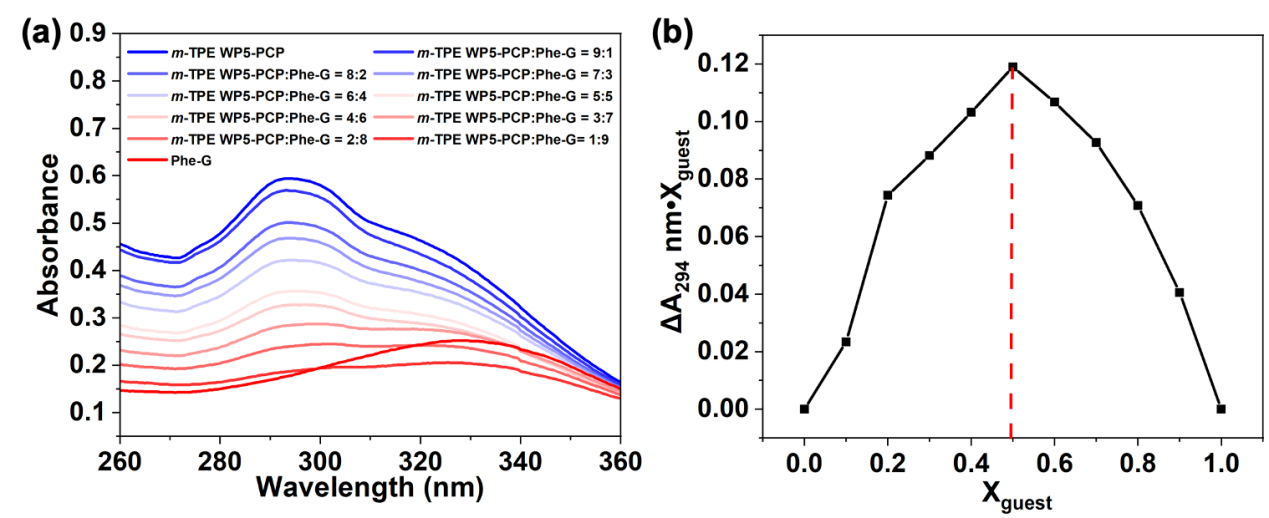


**Figure S28.** (a) The UV/vis spectra of ***m*-TPE WP5-PCP**/**Phe-G** complex ([***m*-TPE WP5-PCP**]+[**Phe-G**] = 0.02 mM) in water. (b) Job’s plot of ***m*-TPE WP5-PCP**/**Phe-G** complex ([***m*-TPE WP5-PCP**]+[**Phe-G**] = 0.02 mM) in water according to the absorbance at 294 nm.

**Dynamic chiral regulation**


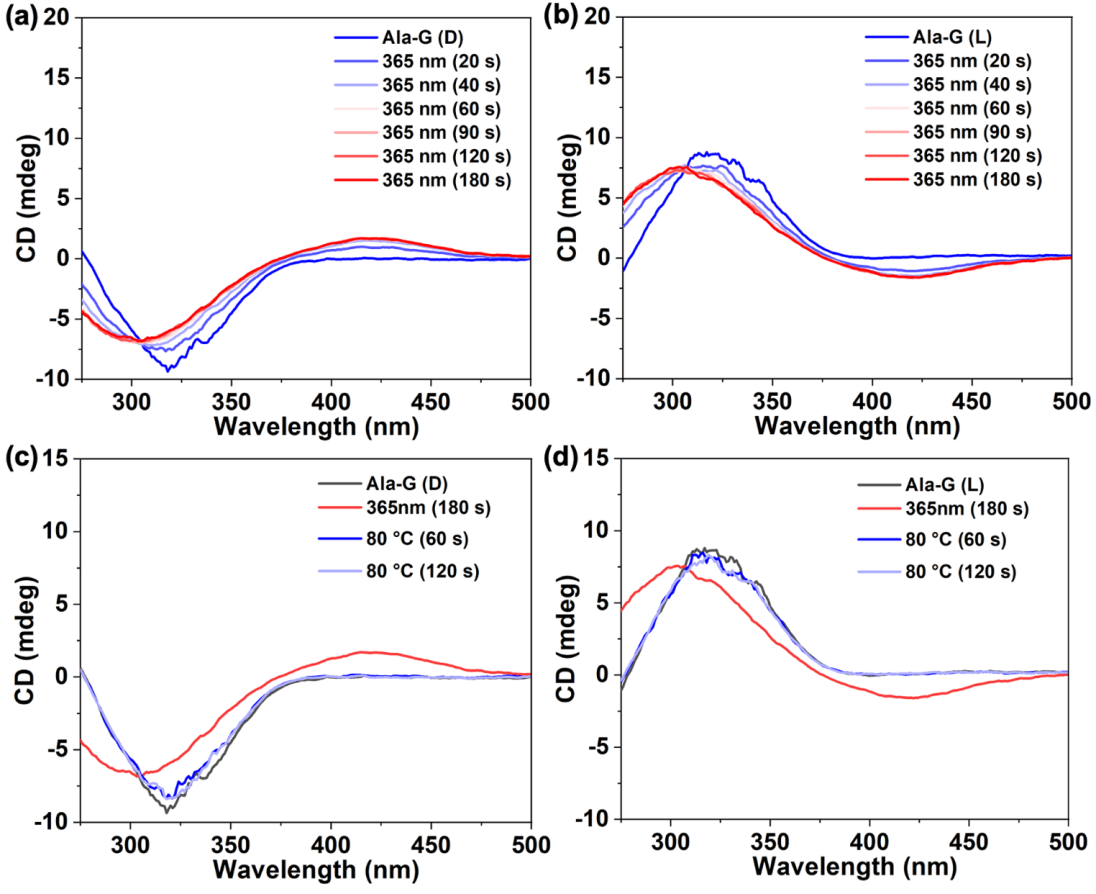


**Figure S29.** (a) Time-dependent CD spectra of **Ala-G** (D) upon irradiation at 365 nm in water. (b) Time-dependent CD spectra of **Ala-G** (L) upon irradiation at 365 nm in water. (c) CD spectral changes of **Ala-G** (D) under irradiation at 365 nm and heating at 80 ˚C in water. (d) CD spectral changes of **Ala-G** (L) under irradiation at 365 nm and heating at 80 ˚C in water.


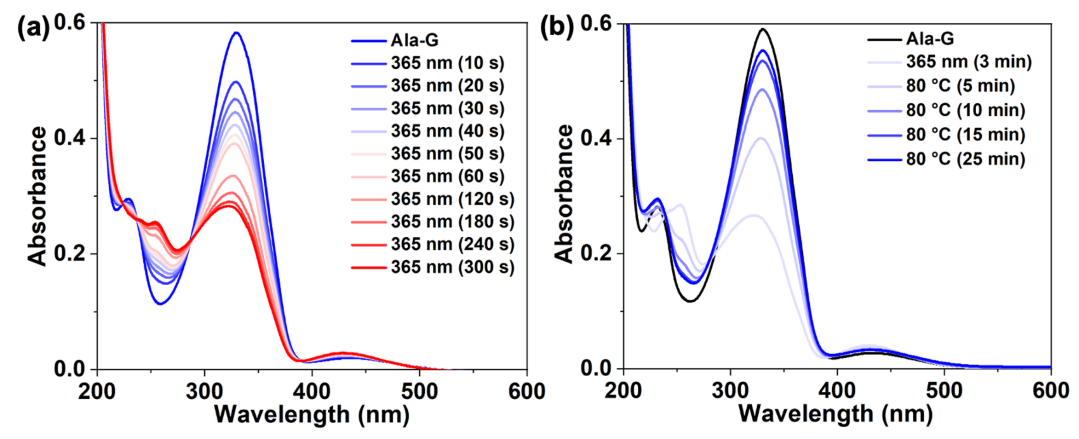


**Figure S30.** (a) Time-dependent UV spectra of **Ala-G** upon irradiation at 365 nm in water. (b) UV spectral changes of **Ala-G** under irradiation at 365 nm and heating at 80 ˚C in water.

Geometry optimizations and vibrational analysis were performed for all structures with Gaussian 16 (C.02) software package using M06-2X exchange-correlation functional,^[S8,S9]^ combined with the def2-SVP basis set.^[S10]^ The counterpoise method of Boys and Bernardi was employed to address the basis set superposition error (BSSE) in the evaluation of interaction energy for host-guest complexes.^[S11]^ Dispersion corrections were taken into account in both geometry optimizations and binding energy calculations, by adopting the D3 version of Grimme’s dispersion with the Becke–Johnson damping function.^[S12]^


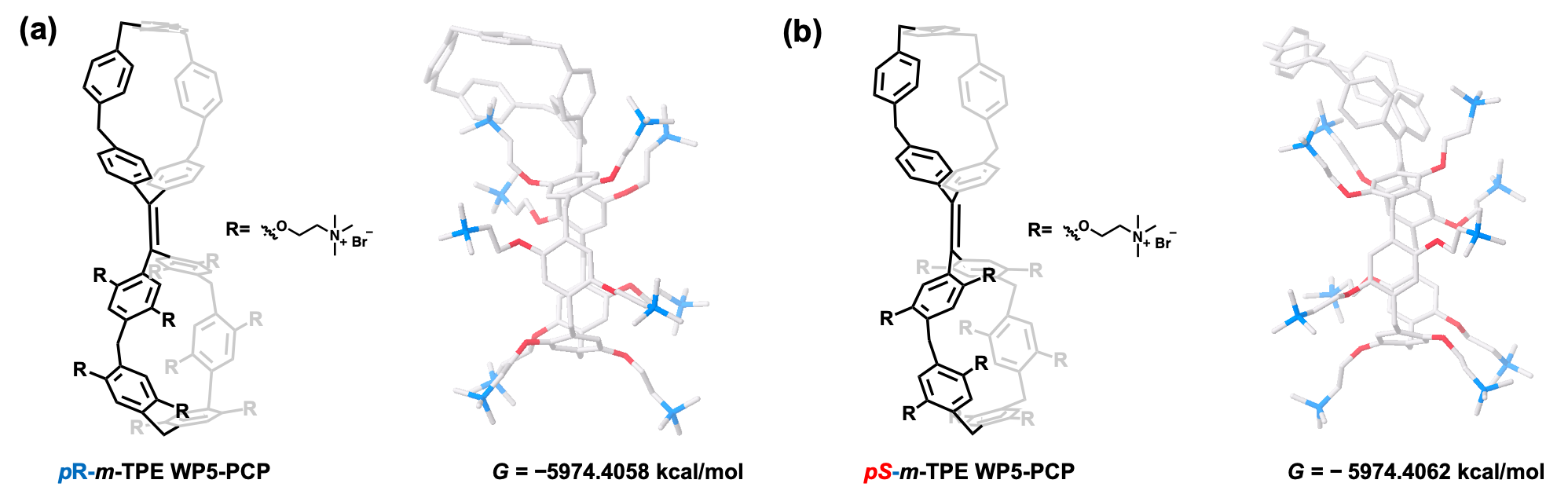


**Figure S31.** Free energies and optimized structures of (a) ***p*R-*m*-TPE WP5-PCP** and (b) ***p*S-*m*-TPE WP5-PCP**. Hydrogens of the complexes are omitted for clarity.

Molecular dynamics (MD) simulations were carried out to obtain possible conformations of the complexes in aqueous solution. All calculations were performed using Materials Studio software. The Forcite module was employed to conduct geometric optimization of the constructed molecular models. The precision was set to "fine", and the COMPASSIII force field was selected. Within the force field type interface, specific force field parameters were assigned to each atom in every molecule. After completing the force field parameterization, charges were assigned using the change using forcefield assigned option. In the Amorphous Cell (AC) module, solution boxes were constructed based on the molar ratios of the solute and various solvents. The systems were hydrated by periodic water box of dimension 50 Å x 50 A x 50 Å and neutralized by counter ion (Br ion). The density of aqueous solution was approximately set to 1.00 g/cm^3^. These solution boxes were then subjected to structural optimization in the Forcite module to minimize energy. Subsequently, a 2 ns NPT (constant number of particles, pressure, and temperature) simulation was performed at 298 K and 1 atm to achieve equilibrium density, followed by a 10 ns NVT simulation to reach final equilibrium.


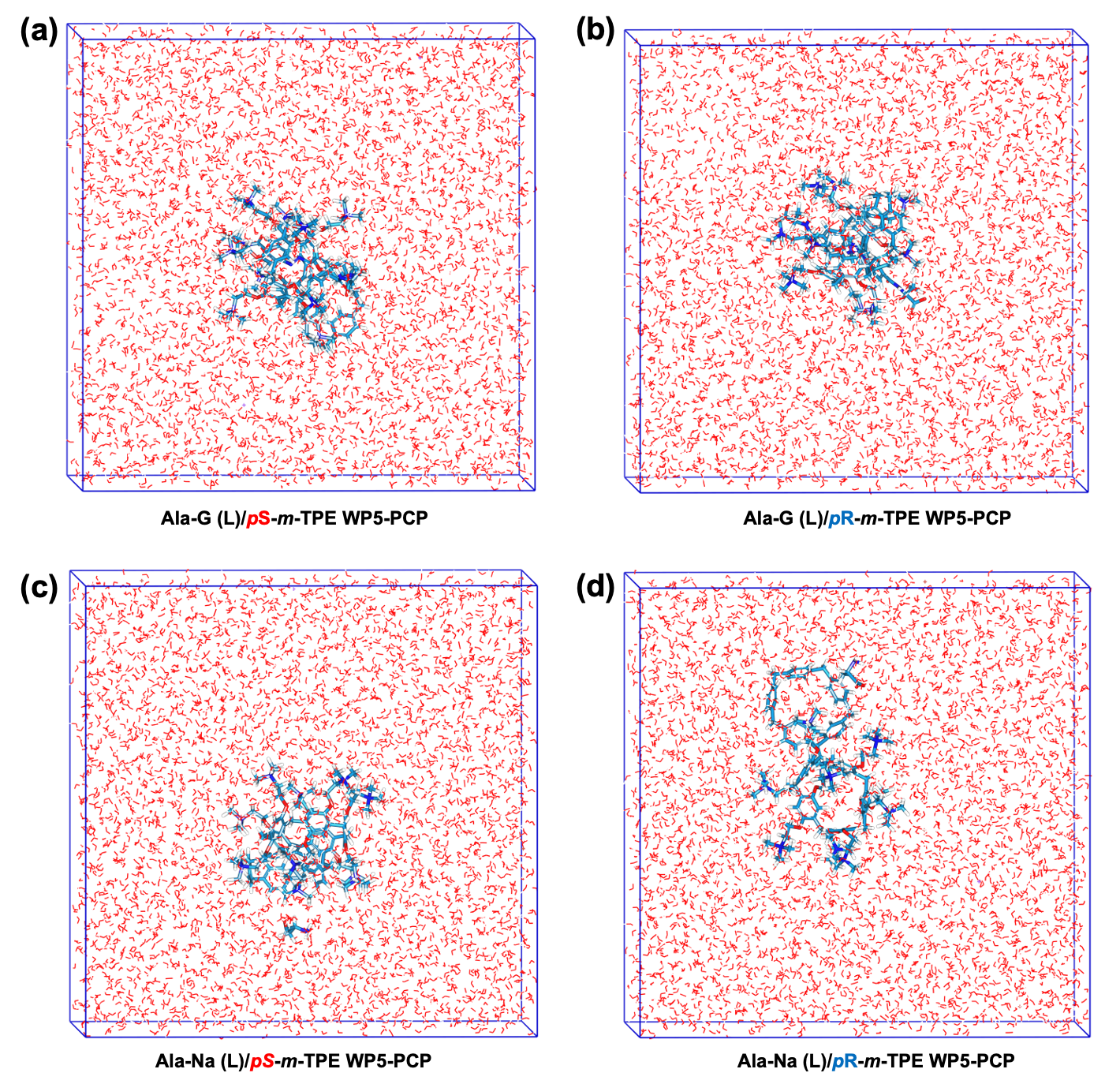


**Figure S32.** Molecular dynamics simulations of (a) **Ala-G** (L)/*p*S*-****m*-TPE WP5-PCP**, (b) **Ala-G** (L)/*p*R*-****m*-TPE WP5-PCP**, (c) **Ala-Na** (L)/*p*S*-****m*-TPE WP5-PCP**, and (d) **Ala-Na** (L)/*p*R*-****m*-TPE WP5-PCP**.

For the tetramer systems, geometry optimizations were performed using the semi-empirical extended tight-binding method (GFN2-xTB),^[S13]^ as implemented in the ORCA 6.0 software package.^[S14]^ This approach was chosen to balance computational efficiency with sufficient accuracy for capturing non-covalent interactions in larger molecular assemblies.


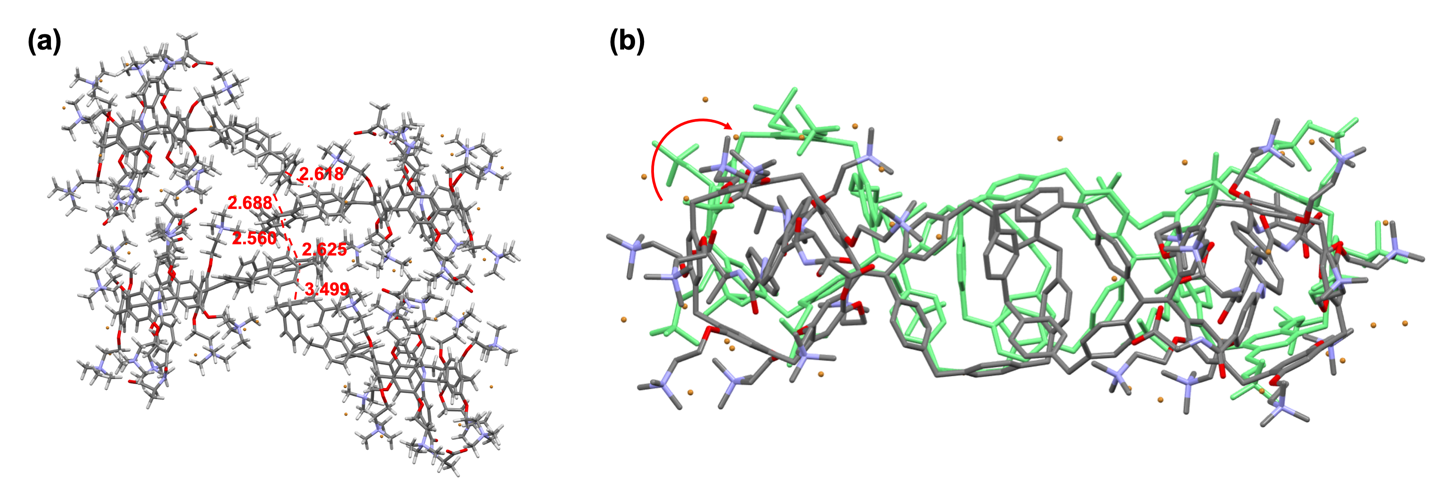


**Figure S33.** Packing models of **Ala-G** (L)/*p*S*-****m*-TPE WP5-PCP** complexes from the front view (a) and the bottom view (b).


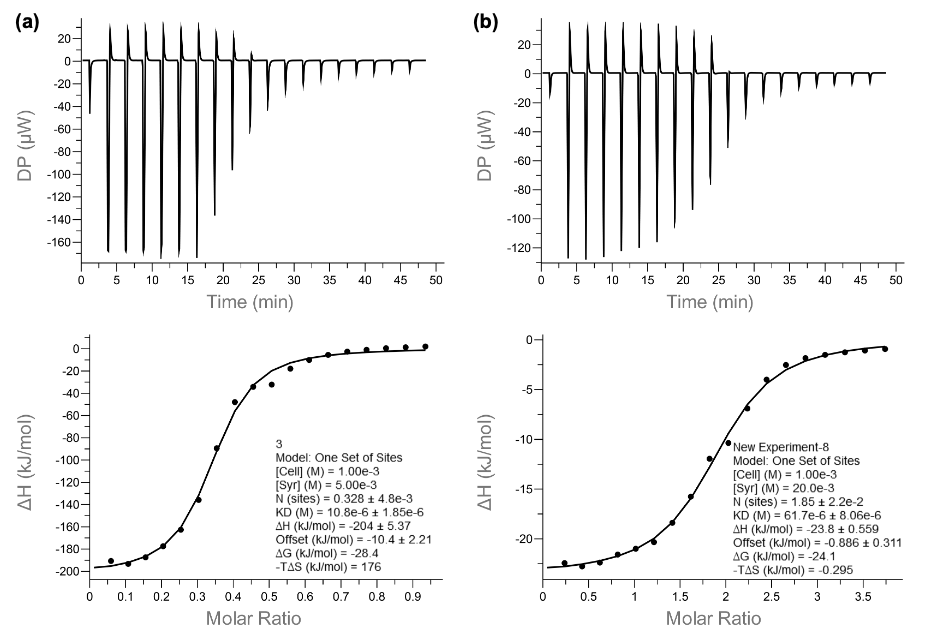


**Figure S34.** Isothermal titration calorimetry (ITC) data of **Ala-G** (L)/***m*-TPE WP5-PCP** (a) and **Ala-Na** (L)/***m*-TPE WP5-PCP** system (b).

**Table S2.** Electronic and Gibbs free energies of optimized structures

| **Entry** | **E_elec_^a,b^** | **ΔE_therm_^a,c^** | **G ^a,d^** |
| --- | --- | --- | --- |
| **pS-m-TPE WP5-PCP** | −5977.0107 | 2.6049 | −5974.4058 |
| **pR-m-TPE WP5-PCP** | −5977.0076 | 2.6014 | −5974.4062 |

a) Energies reported in Table are in atomic units.

b) Single-point electronic energies (*E_elec_*) were calculated at the B3LYP/ma-def2-TZVP level of theory.

c) Thermal corrections were obtained via vibrational analysis at the B3LYP/def2-SVP level.

d) The Gibbs free energy of a given species is obtained by adding the thermal correction to its electronic energy: *G = E_elec_ + ΔE_therm_*.

**Table S3.** Interaction energy for host-guest complexes for host-guest complexes

| **Entry** | **E_int** |
| --- | --- |
| **Ala-G (L)/pS-m-TPE WP5-PCP** | −690.9 |
| **Ala-G (L)/pR-m-TPE WP5-PCP** | −721.5 |
| **Ala-Na (L)/pS-m-TPE WP5-PCP** | −299.7 |
| **Ala-Na (L)/pR-m-TPE WP5-PCP** | −279.3 |

a) All energies are given in kcal/mol.

b) The corrections accounting for the basis set superposition error (BSSE) were obtained for the optimized structures of complexes at the B3LYP/ma-def2-TZVP level of theory.

1. **Supplementary Reference**

[S1] M. R. Pinto, B. M. Kristal, K.S. Schanze, “A water-soluble poly(phenylene ethynylene) with pendant phosphonate groups. synthesis, photophysics, and layer-by-layer self-assembled films” *Langmuir* **2003**, *19*, 6523.

[S2] T. Ogoshi, S. Kanai, S. Fujinami, T. A. Yamagishi, Y. Nakamoto, “*para*-Bridged symmetrical pillar[5]arenes: their lewis acid catalyzed synthesis and host–guest property” *J. Am. Chem. Soc.* **2008***, 130,* 5022.

[S3] X. Tian, M. Zuo, P. Niu, K. Velmurugan, K. Wang, Y. Zhao, L. Wang, X.-Y. Hu, “Orthogonal design of a water-Soluble meso-tetraphenylethene-functionalized pillar[5]arene with aggregation-induced emission property and its therapeutic application” *ACS Appl. Mater. Interfaces* **2021**, *13*, 37466.

[S4] E. Meichsner, I. Nierengarten, M. Holler, M. Chessé, J.-F. Nierengarten, “A fullerene-substituted pillar[5]arene for the construction of a photoactive rotaxane” *Helv. Chim. Acta* **2018**, *101*, e1800059.

[S5] T. Ogoshi, T. Aoki, K. Kitajima, S. Fujinami, T. Yamagishi, Y. Nakamoto, “facile, rapid, and high-yield synthesis of pillar[5]arene from commercially available reagents and its X-ray crystal structure” *J. Org. Chem.* **2011**, *76*, 328.

[S6] P. Liu, Q. Li, H. Zeng, B. Shi, J. Liu, F. Huang, “[1_5_]Paracyclophane and [1_6_]paracyclophane: facile syntheses, crystal structures and selective complexation with cesium cations in the gas phase” *Org. Chem. Front.* **2019**, *6*, 309.

[S7] K. Wang, X. Huang, M. Mohan, K. Zhang, M. Zuo, Y. Shen, Y. Zhao, J. Niemeyer, X.-Y. Hu, “Tetraphenylethylene-embedded [1_5_]paracyclophanes: AIEgen and macrocycle merged novel supramolecular hosts used for sensing Ni^2+^ ions” *Chem. Commun.* **2022**, *58*, 6196.

[S8] M. J. Frisch, G. W. Trucks, H. B. Schlegel, G. E. Scuseria, M. A. Robb, J. R. Cheeseman, et al., Gaussian 16, Revision C.02, Gaussian, Inc., Wallingford, CT, **2019**.

[S9] Y. Zhao, D. G. Truhlar, “The M06 suite of density functionals for main group thermochemistry, thermochemical kinetics, noncovalent interactions, excited states, and transition elements: two new functionals and systematic testing of four M06-class functionals and 12 other functionals.” *Theor. Chem. Account.* **2008**, *120*, 215.

[S10] A. Schäfer, H. Horn, R. Ahlrichs, “Fully optimized contracted Gaussian basis sets for atoms Li to Kr” *J. Chem. Phys.* **1992**, *97*, 2571.

[S11] S. F. Boys, F. Bernardi, “The calculation of small molecular interactions by the differences of separate total energies. Some procedures with reduced errors” *Mol. Phys.* **1970**, *19*, 553.

[S12] S. Grimme, S. Ehrlich, L. Goerigk, “Effect of the damping function in dispersion corrected density functional theory” *J. Comput. Chem*. **2011**, *32*, 1456.

[S13] C. Bannwarth, S. Ehlert, S. Grimme “GFN2-xTB—An accurate and broadly parametrized self-consistent tight-binding quantum chemical method with multipole electrostatics and density-dependent dispersion contributions” *J. Chem. Theory Comput.* **2019**, *15*, 1652.

[S14] F. Neese, Software update: The ORCA program system, version 6.0. *WIREs Comput. Mol. Sci.* **2025**, *15*, e70019.
